# Metabolomic and Transcriptomic Responses of *Rhododendron delavayi* Petals Infected with *Neopestalotiopsis clavispora* at Different Stages

**DOI:** 10.3390/plants15152362

**Published:** 2026-07-31

**Authors:** Yunhong Luo, Su Gong, Yizhen Wang, Fubao Wu, Shanshan Yu, Ximin Zhang

**Affiliations:** 1 School of Life Sciences, Guizhou Normal University, Guiyang 550025, China; luoyunhong_001@163.com (Y.L.); gongsu188@163.com (S.G.); wangyizhen1689@163.com (Y.W.); wufubao101@163.com (F.W.); yushanshan1932@163.com (S.Y.); 2 Key Laboratory of Environment Friendly Management on Alpine Rhododendron Diseases and Pests of Institutions of Higher Learning in Guizhou Province, Guizhou Normal University, Guiyang 550025, China; 3 Engineering Research Center of Carbon Neutrality in Karst Areas, Ministry of Education, Guizhou Normal University, Guiyang 550025, China

**Keywords:** *Rhododendron delavayi*, *Neopestalotiopsis clavispora*, metabolomics, transcriptomics, phloretin, petal blight resistance

## Abstract

Petal blight caused by *Neopestalotiopsis clavispora* infection in *Rhododendron delavayi* petals severely reduces their ornamental value; however, the metabolic and transcriptional responses of petals to this pathogen remain unclear. In this study, we integrated widely targeted metabolomic and transcriptomic analyses to systematically characterize the response mechanisms of *R. delavayi* petals at early (1 day post-infection), middle (2 days post-infection), and late (4 days post-infection) stages of *N. clavispora* infection. A total of 1251 metabolites were identified, and K-means clustering analysis revealed that the differentially accumulated metabolites (DAMs) in Subclass 1 were significantly enriched in flavonoid, phenylpropanoid, and glutathione metabolic pathways. Transcriptomic analysis identified 3744, 6986, and 5407 differentially expressed genes (DEGs) at early, middle and late stages, respectively. Weighted gene co-expression network analysis (WGCNA) indicated that the key hub genes at early, middle, and late stages were mainly involved in maintaining reactive oxygen species (ROS) homeostasis, plant–pathogen interaction signal transduction, and cell wall remodeling, respectively. Joint metabolomic and transcriptomic analysis showed that phenylpropanoid, flavonoid, and glutathione pathways were commonly and significantly enriched. In vitro antifungal assays demonstrated that 2000 mg/L phloretin significantly inhibited mycelial growth of *N. clavispora* and effectively alleviated petal infection. Collectively, *R. delavayi* petals exhibit stage-specific defense strategies against *N. clavispora*: in the early stage, by inducing H_2_O_2_ accumulation and ROS homeostasis; in the middle stage, by activating immune signaling pathways; and in the late stage, by enhancing cell wall remodeling and antioxidant capacity. This study provides a theoretical basis and a candidate compound for green control of petal blight.

## 1. Introduction

Alpine rhododendrons are a group of evergreen or deciduous shrubs distributed in high-altitude mountain environments. They are key components of subalpine ecosystems and play important roles in maintaining biodiversity and ecosystem stability [1,2,3,4]. As world-famous woody ornamental flowers, alpine rhododendrons also significantly promote local tourism economies. For example, the Baili Azalea Scenic Area in Guizhou Province, China, received 7.6976 million tourists in 2024, generating a total tourism revenue of 10.161 billion Yuan [5]. However, petal blight disease caused by *Neopestalotiopsis clavispora* has broken out with increasing frequency in recent years. This pathogen readily infects rhododendron petals and generates extensive brown necrotic lesions, drastically shortening the ornamental lifespan of flowers, and it has become a critical bottleneck restricting the sustainable development of alpine rhododendron tourism and ornamental industries in Guizhou Province. Research on *N. clavispora* in *Rhododendron* species has been reported in multiple cultivars of *Rhododendron hybridum*. Previous studies have characterized the response characteristics of leaves and petals of *R. hybridum* to this pathogen [6,7]. However, studies concerning metabolic alterations and transcriptional regulation in *Rhododendron delavayi* petals following infection by *N. clavispora* are relatively scarce. Therefore, elucidating the molecular and metabolic mechanisms by which rhododendron flowers defend against pathogen infection is essential for the green control of petal blight.

Plants rely on a multi-pathway cooperative network to resist pathogen invasion. Preformed physical barriers (e.g., cell walls and cuticles) constitute the first line of defense against pathogen entry [8,9]. Once the pathogen breaches these physical barriers, pattern recognition receptors (PRRs) on the cell membrane activate pattern-triggered immunity (PTI) [10,11,12], which, in turn, drives the expression of numerous defense-related genes and the accumulation of secondary metabolites. The accumulated secondary metabolites can directly inhibit pathogens, for example, by disrupting the integrity of the pathogen cell membrane [13,14] or interfering with the function of virulence factors [15,16]. In recent years, numerous studies have confirmed that plant immune responses exhibit distinct stage-specific characteristics upon pathogen infection: signal perception at the early stage, metabolic reprogramming at the middle stage, and reinforcement of defensive barriers at the late stage [17,18]. In various plant–pathogen interaction systems, several key metabolites with disease resistance functions have been reported. For instance, infection of *Nicotiana attenuata* leaves by *Alternaria alternata* induces the accumulation of scopoletin and capsidiol [19,20,21]; infection of *Oryza sativa* leaves by *Ustilaginoidea virens* induces the accumulation of 2,4-di-tert-butylphenol (2,4-DTBP) [13]; and infection of *Lilium* leaves by *Botrytis elliptica*, *Botrytis cinerea*, and *Fusarium oxysporum* induces the accumulation of caffeic acid, naringenin, coumarin, and kaempferol [17,22]. These accumulated metabolites enhance plant resistance to pathogens. In *Rhododendron* species, it has been reported that metabolites such as apigenin and naringenin can enhance petal resistance to *Alternaria alternata* and *Neopestalotiopsis clavispora* [23,24,25,26]. However, for alpine rhododendrons, the patterns of metabolite changes in petals after pathogen infection and the underlying transcriptional regulatory networks remain unclear and require further investigation.

*Rhododendron delavayi* is an important dominant species among alpine rhododendrons, and its red flowers also possess extremely high ornamental value within this group. In the Baili Azalea Scenic Area of Guizhou Province, *R. delavayi* is one of the dominant ornamental species [27,28]. However, the occurrence of petal blight seriously affects the ornamental characteristics of this species. Although a range of relevant studies have been published to characterize rhododendron–pathogen interactions at the multi-omics level, Zhou et al. (2022) combined transcriptomic and proteomic profiling to decipher the responses of *R. delavayi* petals to infection [29]. Other researchers integrated transcriptomics and metabolomics to dissect the early and late defense responses of petals from the cultivar *Rhododendron* ‘Xiaotaohong’ following *Alternaria alternata* infection [24,25]. Additional comparative research has uncovered divergent infection responses between petals and leaves in two rhododendron cultivars, namely, ‘Xiaotaohong’ and ‘Yangmeihong’ [6,7]. Furthermore, previous work has clarified the correlation between divergent petal secondary metabolite profiles and antibacterial activity among three alpine rhododendron species: *R. delavayi*, *Rhododendron agastum*, and *Rhododendron irroratum* [23,26]. In addition, multi-omics studies have also been used to reveal the responses of *Rhododendron* species to abiotic stresses such as heat [30,31,32,33], drought [15,34,35,36], and salt [35,37]. Nevertheless, previous studies have mainly focused on leaves or single sampling time points, lacking systematic analyses of petal defense responses across distinct infection stages [6,7].

To investigate the metabolic responses and transcriptional regulation of *R. delavayi* after infection with *N. clavispora*, we used the petals as materials and combined transcriptomic and widely targeted metabolomic approaches. This study aimed to identify the major metabolic pathways in *R. delavayi* petals following *N. clavispora* infection, analyze the transcriptional regulatory mechanisms underlying these responses, and preliminarily screen for secondary metabolites with inhibitory activity against *N. clavispora*. The results of this study provide important insights into the transcriptional and metabolic defense mechanisms of *R. delavayi* in response to *N. clavispora* infection.

## 2. Results

### 2.1. Effects of N. clavispora Infection on the Phenotype and Lesion Area of R. delavayi Petals

**Figure 1:**
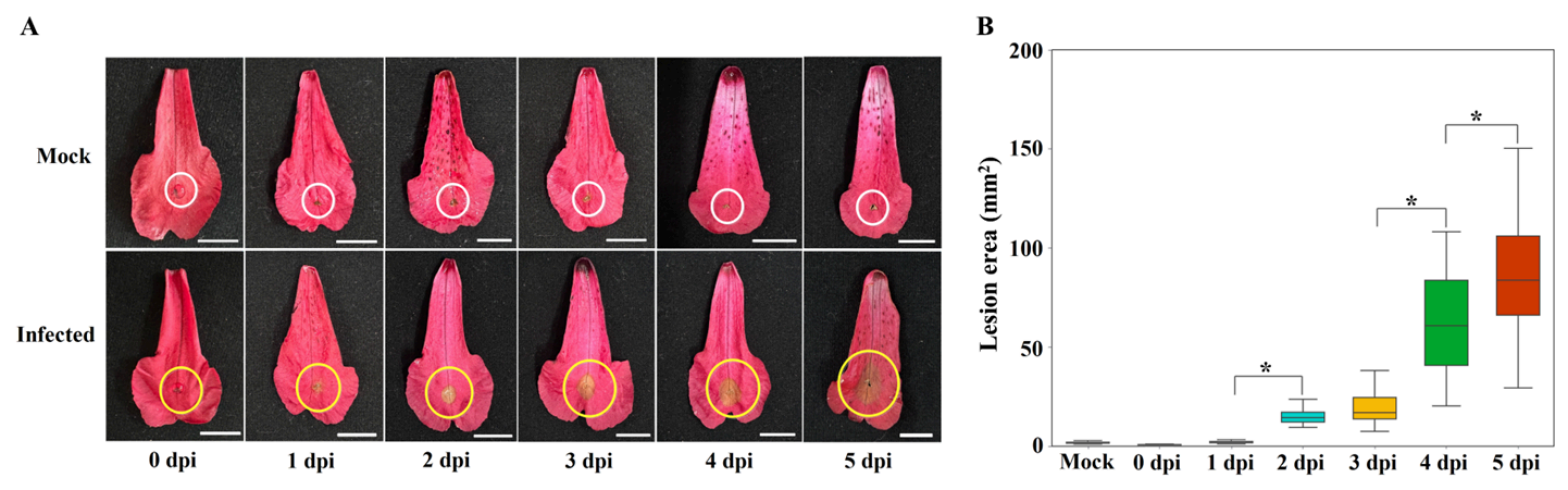
Phenotype and lesion area of *R. delavayi* petals after infection with *N. clavispora*. (**A**) Lesion phenotypes at 0, 1, 2, 3, 4, and 5 dpi (days post-inoculation). Mock and Infected indicate the control group treated with sterile water (white circles) and the treatment group inoculated with mycelial suspension (yellow circles), respectively. Scale bar = 10 mm. (**B**) Lesion areas at 0, 1, 2, 3, 4, and 5 dpi. * indicates significant difference at *p* < 0.05.

To analyze the response of *R. delavayi* petals to *N. clavispora* infection, the lesion area was dynamically monitored from 0 to 5 days post-infection (dpi). The results showed that at 0 dpi, both the treatment group (inoculated with mycelial suspension) and the control group (inoculated with sterile water) were clearly visible on the petals (Figure 1A). At 1 dpi, no symptoms were observed in the control group, whereas brown rot lesions appeared in the treatment group (Figure 1A), with an average lesion area of 2.03 mm^2^, which was significantly smaller than that at 2 dpi (14.95 mm^2^) (Figure 1B). At 3 dpi, the lesion area (20.22 mm^2^) was not significantly different from that at 2 dpi but was significantly smaller than that at 4 dpi (62.43 mm^2^) (Figure 1B). By 5 dpi, the lesion area expanded to 88.48 mm^2^, while the control petals remained asymptomatic throughout. These results indicate that *N. clavispora* infection leads to a gradual increase in the lesion area on *R. delavayi* petals over time.

### 2.2. Hydrogen Peroxide (H_2_O_2_) Accumulation in R. delavayi Petals Induced by N. clavispora Infection

**Figure 2:**
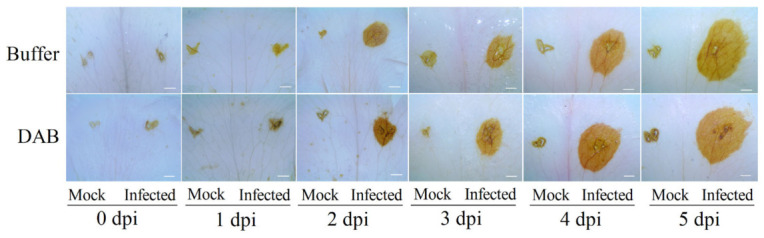
DAB staining of *R. delavayi* petals at different time points after inoculation with *N. clavispora*. DAB, 3,3’-diaminobenzidine. Buffer indicates the control group without DAB staining, and DAB indicates the staining group with DAB added. Mock and Infected represent the control group treated with sterile water and the treatment group inoculated with mycelial suspension, respectively. Scale bar = 10 mm.

Reactive oxygen species (ROS) act as both signaling messengers and defense triggers during plant immune responses. To characterize the dynamic pattern of H_2_O_2_ accumulation upon *N. clavispora* infection, petals harvested at 0–5 dpi were subjected to DAB histochemical staining (Figure 2). At 0 dpi, only faint brown precipitates (the marker of H_2_O_2_) were detected on both mock and infected petals. Relative to the 0 dpi samples, infected petals displayed moderately increased brown staining at 1 dpi, with the most prominent H_2_O_2_ accumulation observed at 2 dpi, demonstrating that pathogen colonization triggered a rapid H_2_O_2_ burst in petal tissues. From 3 to 5 dpi, however, the intensity of brown precipitates gradually faded even as necrotic lesion size expanded continuously, indicating a progressive decline in H_2_O_2_ abundance at the late infection stages.

### 2.3. Identification of Metabolites and Analysis of Differentially Accumulated Metabolites (DAMs)

**Figure 3:**
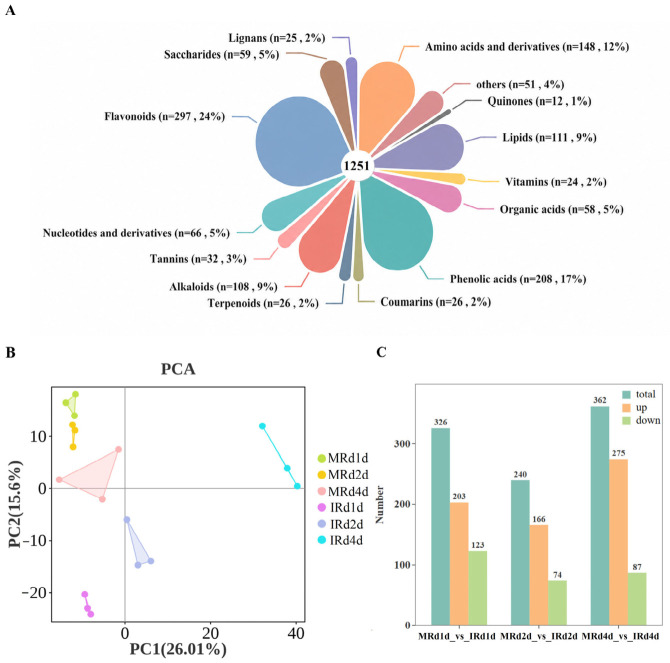
Metabolite profiles of *R. delavayi* petals induced by *N. clavispora* infection at the early (1 dpi), middle (2 dpi), and late (4 dpi) stages. (**A**) Classification and composition of all identified metabolites; (**B**) principal component analysis (PCA) of all identified metabolites, where MRd and IRd represent petal samples treated with sterile water and mycelial suspension, respectively; and (**C**) number of differentially accumulated metabolites (DAMs) identified, with orange bars indicating up-accumulated DAMs and green bars indicating down-accumulated DAMs. MRd1d, MRd2d and MRd4d represent the control groups of *R. delavayi* petals at 1, 2 and 4 days after inoculation with *N. clavispora*, respectively. IRd1d, IRd2d and IRd4d represent the inoculated treatment groups of *R. delavayi* petals at 1, 2, 3 and 4 days after inoculation with *N. clavispora*, respectively.

Based on the lesion area and H_2_O_2_ accumulation dynamics, samples from the control and treatment groups at early (1 dpi), middle (2 dpi), and late (4 dpi) infection stages were selected for widely targeted metabolomic analysis. The total ion current (TIC) chromatograms in positive and negative ion modes showed high overlap among samples (Figure S1), indicating good reproducibility among biological replicates and stable instrument performance. Using a self-built metabolite database, a total of 1251 metabolites were identified, which could be classified into 17 categories. Among all identified metabolites, flavonoids (24%), phenolic acids (17%), and amino acids and their derivatives (12%) accounted for relatively high proportions (Figure 3A). Principal component analysis (PCA) of all identified metabolites showed that PC1 and PC2 explained 26.01% and 15.60% of the total variance, respectively. Samples within each group clustered tightly, while clear separation was observed between groups, indicating that infection induced significant changes in petal metabolites (Figure 3B).

Metabolites between the treatment and control groups at early (1 dpi), middle (2 dpi), and late (4 dpi) stages were screened using the following criteria: VIP > 1 and FC ≥ 2. Compared with the control group, 326 DAMs (203 upregulated and 123 downregulated) were identified at the early stage (Figure 3C; Table S1); 240 DAMs (166 upregulated and 74 downregulated) at the middle stage (Figure 3C; Table S2); and 362 DAMs (275 upregulated and 87 downregulated) at the late stage (Figure 3C; Table S3). KEGG enrichment analysis of all DAMs showed that early-stage DAMs were significantly enriched in seven pathways, including flavone and flavanols biosynthesis, phenylpropanoid biosynthesis, and flavonoid biosynthesis (Figure S2A); middle-stage DAMs were significantly enriched in seven pathways, including flavonoid biosynthesis and phenylpropanoid biosynthesis (Figure S2B); and late-stage DAMs were significantly enriched in eight pathways, including flavone and flavanols biosynthesis, flavonoid biosynthesis, phenylpropanoid biosynthesis, and glutathione metabolism (Figure S2C).

**Figure 4:**
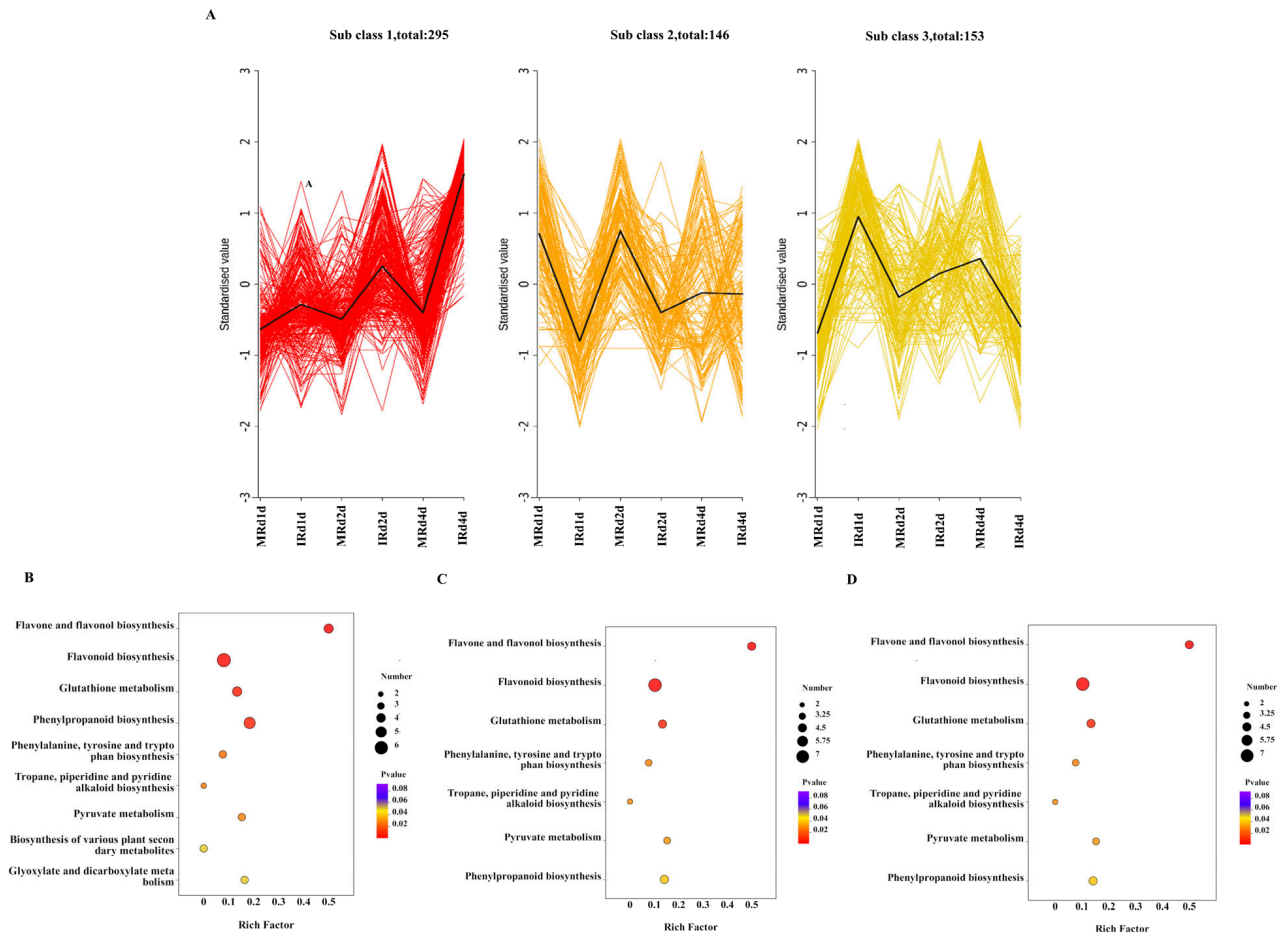
K-means clustering analysis and KEGG enrichment analysis of differentially accumulated metabolites (DAMs) in *R. delavayi* petals at the early (1 dpi), middle (2 dpi), and late (4 dpi) stages of *N. clavispora* infection. (**A**) K-means clustering analysis of DAMs. The x-axis indicates sample groups, and the y-axis indicates the normalized relative content of metabolites. MRd and IRd represent petal samples treated with sterile water and mycelial suspension, respectively. (**B**–**D**) represent the significantly enriched KEGG pathways (*p* < 0.05) of DAMs belonging to Subclass 1 at 1, 2, and 4 dpi, respectively. The x-axis represents the enrichment factor, and the y-axis represents the enriched pathways.

To analyze metabolites associated with infection progression, all DAMs from the three time points were subjected to K-means clustering, yielding three subclasses: Subclass 1 (295 DAMs), Subclass 2 (146 DAMs), and Subclass 3 (153 DAMs) (Figure 4A; Table S4). Considering that pathogen infection induces the accumulation of plant secondary metabolites involved in defense responses, enrichment analysis was performed on DAMs in Subclass 1 (upregulated after infection) (Figure 4B). The results showed that DAMs at the early, middle, and late stages were all significantly enriched in pathways such as flavone and flavanols biosynthesis, flavonoid biosynthesis, glutathione metabolism, and phenylpropanoid biosynthesis.

### 2.4. Transcriptome Sequencing and Analysis of Differentially Expressed Genes (DEGs)

**Figure 5:**
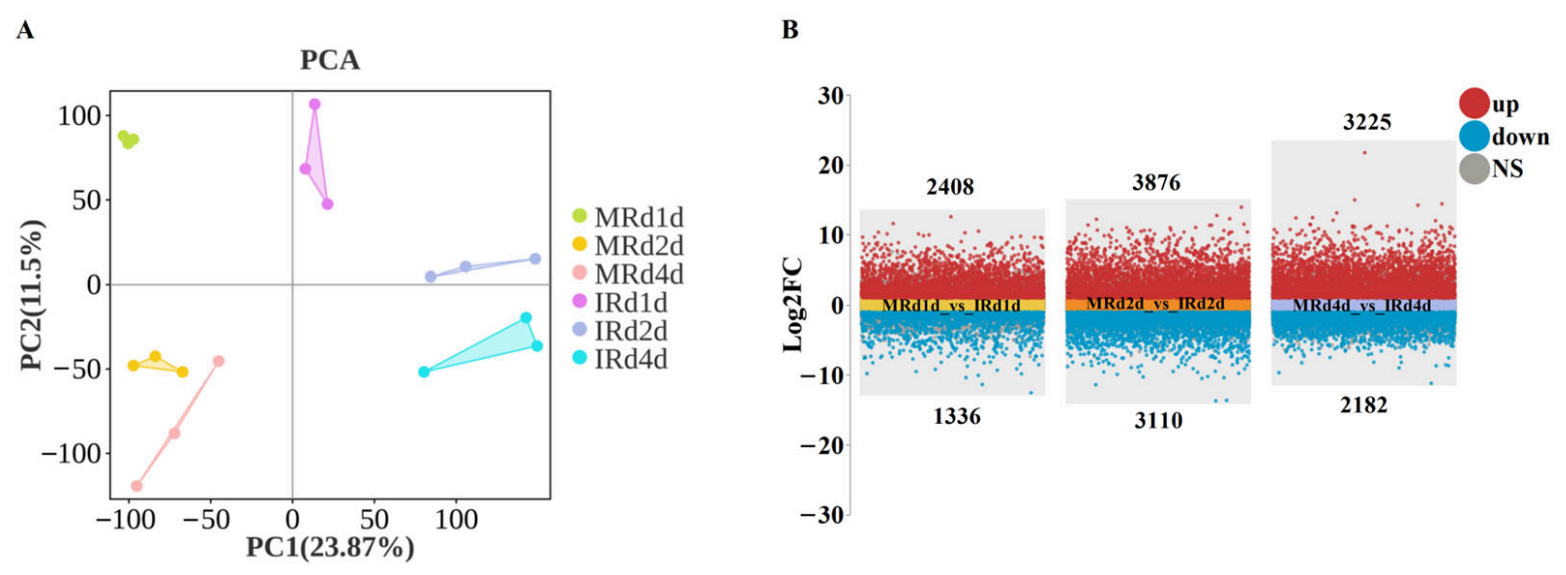
Gene expression profiles of *R. delavayi* petals at the early (1 dpi), middle (2 dpi), and late (4 dpi) stages of *N. clavispora* infection. (**A**) Principal component analysis (PCA) of all detected genes; MRd and IRd represent petal samples treated with sterile water and mycelial suspension, respectively; (**B**) volcano plots of differentially expressed genes (DEGs), with red and blue dots indicating upregulated and downregulated DEGs, respectively.

To explore the gene expression response patterns of *R. delavayi* petals after pathogen infection, transcriptomic analysis was performed on samples from the three time points described above. After quality control, more than 38.86 million clean reads were obtained per sample, with an average GC content of approximately 47%, Q30 ≥ 97.70%, and a mapping rate to the reference genome ranging from 88.93% to 95.92% (Table S5). PCA of all detected genes showed that PC1 and PC2 explained 23.87% and 11.50% of the total variance, respectively (Figure 5A). Biological replicate samples within each treatment clustered tightly, indicating good reproducibility; the control and treatment groups were clearly separated, indicating that *N. clavispora* infection significantly altered the gene expression patterns of *R. delavayi* petals.

DEGs were screened using the following criteria: |log_2_ fold change| > 1 and FDR < 0.05. Compared with the control group, 3744 DEGs (2408 upregulated and 1336 downregulated) were identified at the early stage (Figure 5B; Table S6); 6986 DEGs (3876 upregulated and 3110 downregulated) at the middle stage (Figure 5B, Table S7); and 5407 DEGs (3225 upregulated and 2182 downregulated) at the late stage (Figure 5B; Table S8). Subsequently, Gene Ontology (GO) and Kyoto Encyclopedia of Genes and Genomes (KEGG) enrichment analyses were performed on all DEGs. GO enrichment showed that at the early stage, DEGs were significantly enriched in molecular function (MF) terms such as oxidoreductase activity and catalytic activity; in cellular component (CC) terms such as plasma membrane and cell wall; and in biological process (BP) terms such as response to stimulus, response to chemical, and response to organic substance (Figure S3A). At the middle stage, DEGs were significantly enriched in MF terms including catalytic activity, protein kinase activity, and transferase activity; in CC terms including membrane and endoplasmic reticulum lumen; and in BP terms including defense response, response to stimulus, phosphorylation, and response to biotic stimulus (Figure S3B). At the late stage, DEGs were significantly enriched in MF terms including oxidoreductase activity, protein kinase activity, and transferase activity; in CC terms including cell–cell junction and plasmodesma; and in BP terms including defense response, glutathione metabolic process, and hormone-mediated signaling pathway (Figure S3C). KEGG enrichment showed that early-stage DEGs were significantly enriched in 24 pathways, including plant hormone signal transduction, phenylpropanoid biosynthesis, plant–pathogen interaction (e.g., upregulated genes encoding CNGCs (*Rhdel01G0286600* and *novel4328*), CALM (*Rhdel02G0004700*, *Rhdel04G0332300*, and *novel2645*), CML (*Rhdel02G0061700*, *Rhdel13G0125300*, *Rhdel11G0128200*, *Rhdel10G0048300*, and *Rhdel09G0038900*), CDPK (*novel1630*, *Rhdel03G0075400*, and *Rhdel12G0202100*), Rboh (*Rhdel02G0322900*, *Rhdel08G0252500*, and *Rhdel02G0155400*)), and flavonoid biosynthesis (Figure S3D; Table S9). Middle-stage DEGs were significantly enriched in 28 pathways, including plant hormone signal transduction, biosynthesis of secondary metabolites, and plant–pathogen interaction (e.g., genes encoding FLS2 (*Rhdel01G0251200*, *Rhdel02G0071200*, and *Rhdel07G0134400*), BAK1 (*Rhdel01G0361000*, *Rhdel13G0185600*, and *novel6322), MPK3/6 (Rhdel01G0294100*), WRKY22 (*Rhdel13G0142100* and *Rhdel13G0226500*), and WRKY25/33 (*Rhdel01G0319800* and *Rhdel13G0232300*)) (Figure S3E; Table S10). Late-stage DEGs were significantly enriched in 23 pathways for flavonoid biosynthesis, biosynthesis of secondary metabolites, plant hormone signal transduction, and phenylpropanoid biosynthesis (Figure S3F; Table S11).

### 2.5. Weighted Gene Co-Expression Network Analysis (WGCNA) of DEGs

**Figure 6:**
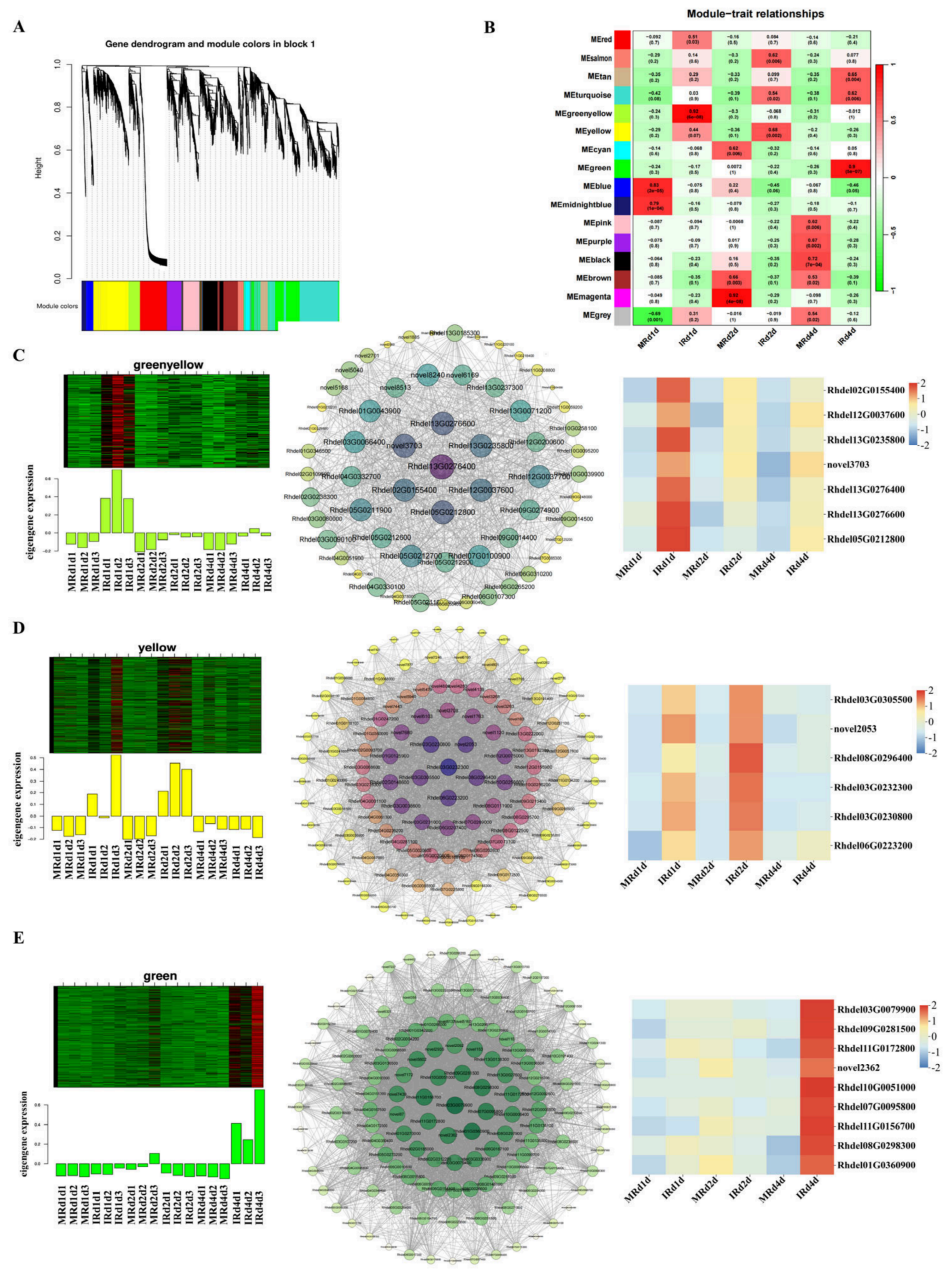
Weighted gene co-expression network analysis (WGCNA) of differentially expressed genes in *R. delavayi* petals at the early (1 dpi), middle (2 dpi), and late (4 dpi) stages of *N. clavispora* infection. (**A**) Gene dendrogram obtained by hierarchical clustering, with module colors shown at the bottom. (**B**) Correlations between modules and traits. Each row corresponds to a module, and each column corresponds to a sample. The numbers in the cells and those in parentheses indicate correlation coefficients and significance levels, respectively. (**C**–**E**) represent the most significant co-expression network diagrams for the early (greenyellow module), middle (yellow module), and late (green module) infection stages, respectively. Left panels: co-expressed genes; middle panels: interaction network of co-expressed genes; and right panels: heatmaps of hub genes, in which red indicates upregulated expression, and blue indicates downregulated expression.

To screen for hub genes, WGCNA was performed based on all DEGs, resulting in 16 co-expression modules (Figure 6A). Module trait correlation analysis showed that the greenyellow module was significantly positively correlated with the early infection stage (*p* = 6 × 10^−8^; *R* = 0.92). The yellow and green modules were significantly positively correlated with the middle (*p* = 0.002; *R* = 0.68) and late (*p* = 5 × 10^−7^; *R* = 0.90) infection stages, respectively (Figure 6B).

Hub genes in the greenyellow, yellow, and green modules were further screened. In the greenyellow module, seven hub genes were identified, including three encoding anionic peroxidase genes (*Rhdel13G0276600*, *Rhdel05G0212800,* and *novel3703*), one cationic peroxidase 1 gene (*Rhdel13G0276400*), one NAD(P)H dehydrogenase gene (*Rhdel02G0155400*), one gibberellin 2-beta-dioxygenase gene (*Rhdel13G0235800*), and one stemmadennine O-acetyltransferase gene (*Rhdel12G0037600*). These genes were specifically upregulated at the early infection stage (Figure 6C). In the yellow module, six hub genes were identified, including three encoding plant receptor-like kinase genes (*Rhdel03G0230800*, *Rhdel03G0232300*, and *novel2053*), one 2-oxoglutarate/Fe(II)-dependent oxygenase gene (*Rhdel03G0305500*), one serine/threonine protein kinase gene (*Rhdel08G0296400*), and one WRKY27 transcription factor (*Rhdel06G0223200*). These genes were specifically upregulated at the middle infection stage (Figure 6D). In the green module, nine hub genes were identified, including three encoding oxidoreductase genes (*Rhdel10G0051000*, *Rhdel08G0298300*, and *Rhdel11G0156700*), one cytokinin response factor 4 (CRF4) transcription factor (*Rhdel09G0281500*), one protein kinase gene (*Rhdel01G0360900*), one E3 ubiquitin ligase gene (*Rhdel03G0079900*), one cellulose synthase-like gene (*Rhdel07G0095800*), one multidomain transmembrane protein gene (*Rhdel11G0172800*), and one receptor-like kinase gene (*novel2362*). These genes were specifically upregulated at the late infection stage (Figure 6E).

### 2.6. Integrated Analysis of Transcriptome and Metabolome

**Figure 7:**
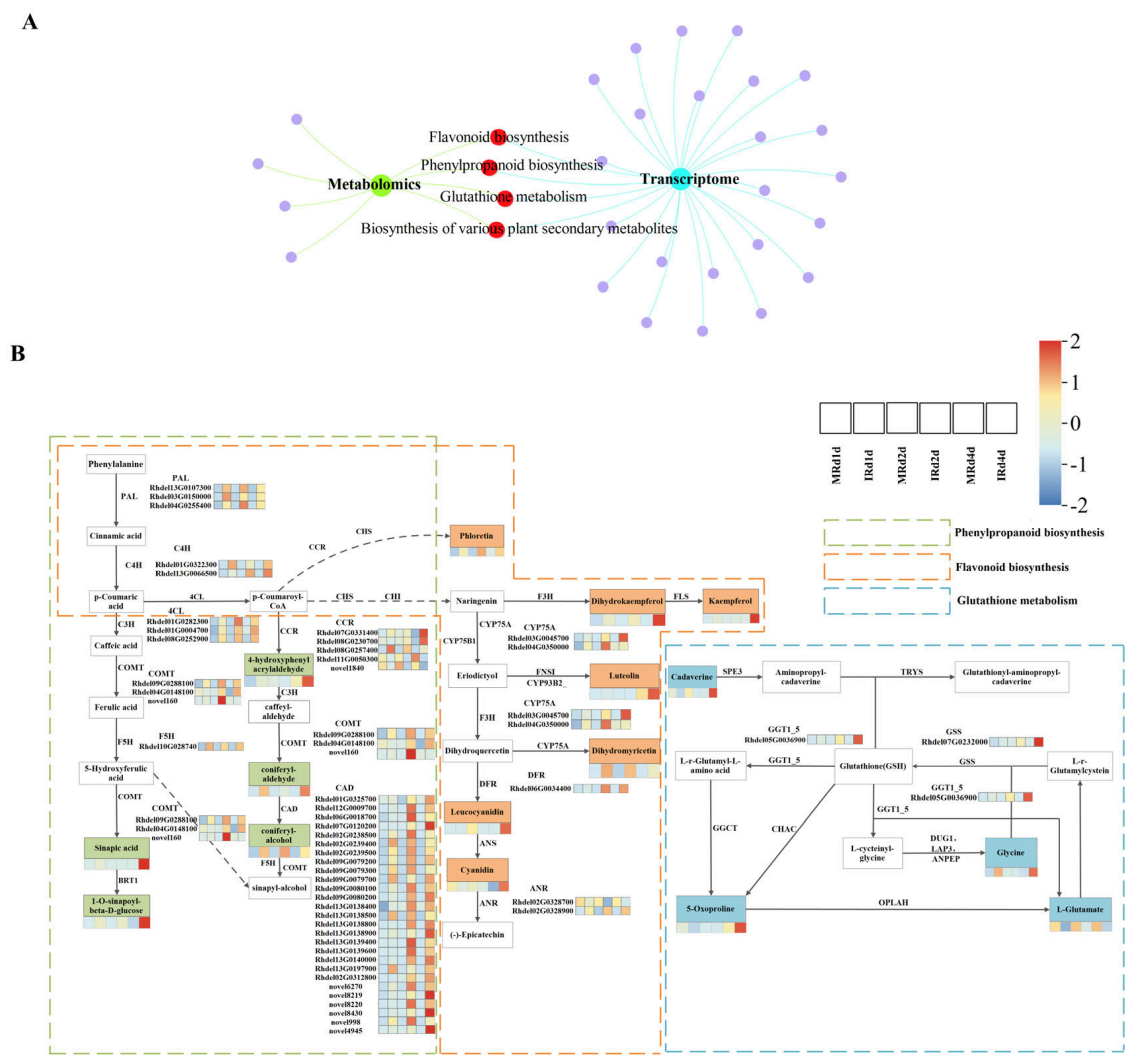
Integrated analysis of transcriptome and metabolome. (**A**) Venn diagram of KEGG enrichment pathways of differentially accumulated metabolites in Subclass 1 from K-means clustering across the three time points and differentially expressed genes across the three time points. (**B**) Partial regulatory network of genes and metabolites in the phenylpropanoid, flavonoid, and glutathione metabolic pathways. Green, orange, and blue dashed boxes represent the phenylpropanoid biosynthesis pathway, flavonoid biosynthesis pathway, and glutathione metabolism pathway, respectively. Heatmaps of metabolites are displayed below their names, and heatmaps of gene expression are shown adjacent to the gene names; color scales are indicated in the upper right corner. PAL, phenylalanine ammonia-lyase; C4H, trans-cinnamate 4-monooxygenase; C3H, p-coumarate 3-hydroxylase; COMT, caffeic acid 3-O-methyltransferase/acetylserotonin O-methyltransferase; F5H, ferulate-5-hydroxylase; BRT1, sinapate 1-glucosyltransferase; 4CL,4-coumarate--CoA ligase; CCR, cinnamoyl-CoA reductase; CHS, chalcone synthase; CHI, chalcone isomerase; CAD, cinnamyl-alcohol dehydrogenase; F3H, naringenin 3-dioxygenase; FLS, flavonol synthase; CYP75A, flavonoid 3’,5’-hydroxylase; CYP75B1, flavonoid 3’-monooxygenase; DFR, bifunctional dihydroflavonol 4-reductase/flavanone 4-reductase; ANS, anthocyanidin synthase; ANR, anthocyanidin reductase; FNSI, flavone synthase I; CYP93B2_16, flavone synthase II.

To systematically dissect the metabolomic and transcriptomic responses of *R. delavayi* petals to *N. clavispora* infection, integrative analysis of metabolomic and transcriptomic data was performed. KEGG enrichment analysis was conducted on DAMs in Subclass 1 (early, middle, and late stages) from K-means clustering (Figure 4A) and on DEGs at the three stages (Figure S3; Tables S12–S14). Venn diagram analysis revealed that the pathways flavonoid biosynthesis, phenylpropanoid biosynthesis, glutathione metabolism, and biosynthesis of various plant secondary metabolites were significantly co-enriched at all three infection stages (Figure 7A). This indicates that metabolites and genes in these pathways may play important roles in the response to *N. clavispora* infection.

In the phenylpropanoid biosynthesis pathway, genes encoding PAL (*Rhdel13G0107300*, *Rhdel03G0150000*, and *Rhdel04G0255400*), CYP73A (*Rhdel01G0322300*, *Rhdel13G0066500*), COMT (*Rhdel09G0288100* and *Rhdel04G0148100*), and F5HR (*Rhdel10G0287400*) were significantly upregulated after *N. clavispora* infection, accompanied by significant accumulation of sinapic acid and 1-O-sinapoyl-beta-D-glucose (Figure 7B). In addition, genes encoding 4CL (*Rhdel01G282300*, *Rhdel01G0004700*, and *Rhdel08G0252900*), CCR (*Rhdel07G0331400*, *Rhdel08G0230700*, and *Rhdel08G0257400*), and CAD (*Rhdel01G0325700*, *Rhdel12G0009700*, *Rhdel06G0018700*, *Rhdel02G0239400*, *Rhdel02G0239500*, *Rhdel09G0079200*, and *Rhdel09G0079300*) were significantly upregulated after infection, accompanied by significant accumulation of 4-hydroxyphenyl acrylaldehyde, coniferyl-aldehyde, and coniferyl-alcohol (Figure 7B).

In the flavonoid biosynthesis pathway, genes encoding PAL (*Rhdel13G0107300*, *Rhdel03G0150000*, and *Rhdel04G0255400*) and CYP73A (*Rhdel01G0322300* and *Rhdel13G0066500*) were significantly upregulated after *N. clavispora* infection, accompanied by significant accumulation of phloretin, dihydrokaempferol, and kaempferol (Figure 7B). Moreover, genes encoding CYP75A (*Rhdel03G0045700* and *Rhdel04G0350000*), DFR (*Rhdel06G0034400*), and ANR (*Rhdel02G0328900*) were significantly upregulated after infection, accompanied by significant accumulation of luteolin, dihydromyricetin, leucocyanidin, and cyanidin (Figure 7B).

In the glutathione metabolism pathway, genes encoding GGT15 (*Rhdel05G0036900*) and GSS (*Rhdel07G0232000*) were significantly upregulated after *N. clavispora* infection, accompanied by significant accumulation of cadaverine, glycine, and 5-oxoproline (Figure 7B).

To further quantitatively dissect the potential regulatory links between key genes and metabolites and verify the effect of transcriptional variation on metabolite accumulation, Pearson correlation coefficients were calculated between the expression levels of hub genes screened via WGCNA and the relative contents of differentially accumulated metabolites. Gene–metabolite pairs satisfying |r| ≥ 0.70 and *p* < 0.05 were retained to construct the correlation heatmap (Figure S5). Hierarchical clustering revealed that all molecules were divided into two distinct modules. L-glutamic acid exhibited significant negative correlations with most tested genes. In contrast, metabolites involved in the phenylpropanoid and flavonoid pathways, including phloretin, coniferyl alcohol, dihydromyricetin, cyanidin, kaempferol, and sinapic acid, showed strong positive correlations (r ≥ 0.70) with the majority of disease-resistant structural genes. These results indicate that the upregulation of the above hub genes under pathogen stress markedly facilitates the biosynthesis and accumulation of downstream defensive flavonoid and phenylpropanoid secondary metabolites. This finding statistically confirms the regulatory patterns obtained from the integrated enrichment analysis of transcriptome and metabolome data and further supports the regulatory relationship of genes governing metabolite accumulation.

### 2.7. Inhibitory Effects of Exogenous Metabolites on Mycelial Growth of N. clavispora

**Figure 8:**
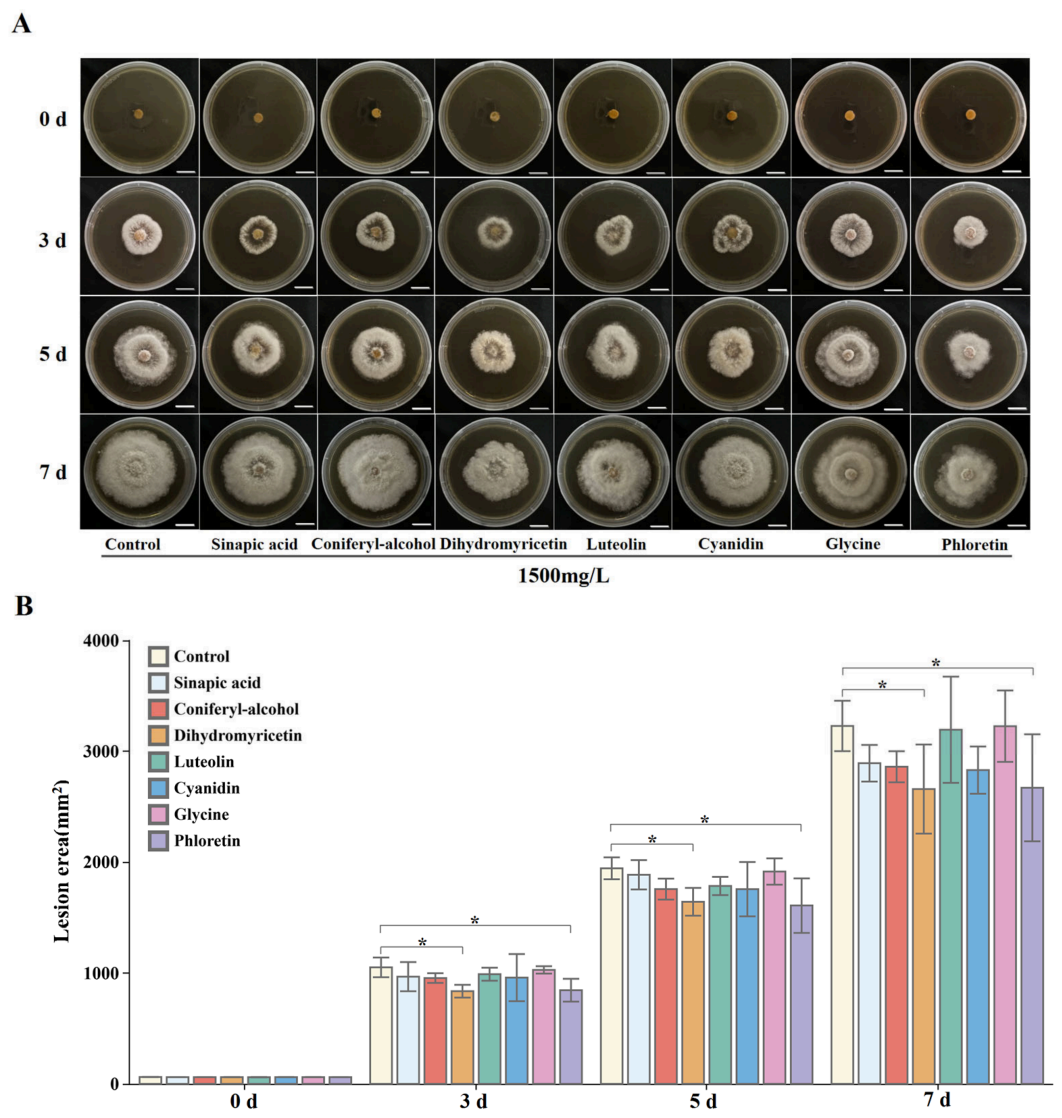
Inhibition of mycelial growth of *N. clavispora* by the screened metabolites. (**A**) Mycelial growth phenotypes of *N. clavispora.* Control: group treated with 1% (*v*/*v*) NaOH solution. Scale bar = 10 mm. (**B**) Colony areas of *N. clavispora* at 0, 3, 5, and 7 d (day). * indicates significant difference at *p* < 0.05.

Among the 16 upregulated secondary metabolites identified by the integrated analysis, kaempferol, cyanidin, dihydromyricetin, luteolin, phloretin, sinapic acid, 4-hydroxyphenyl acrylaldehyde, coniferyl-aldehyde, coniferyl-alcohol, and glycine have been reported to possess antifungal activity (Table 1). To eliminate the interference of solvents on the evaluation of antifungal activity, the effects of 1% DMSO and 1% NaOH on the mycelial growth of *N. clavispora* were determined. The average colony areas of blank control groups at 1, 2 and 4 d were 1187.40 mm^2^, 1978.40 mm^2^ and 3460.68 mm^2^, respectively, while those of solvent control groups were 1051.19 mm^2^, 1942.18 mm^2^ and 3227.53 mm^2^. Statistical analysis revealed no significant differences between the two groups (Table S15), demonstrating that the solvent concentrations adopted in this study exerted no adverse impacts on the normal growth of *N. clavispora*. Therefore, the antifungal effects observed in subsequent assays can be exclusively attributed to the tested metabolites. Since kaempferol was tested for antifungal activity in our previous study, the present study performed a preliminary antifungal activity screening for dihydromyricetin, luteolin, phloretin, cyanidin, glycine, coniferyl-alcohol, and sinapic acid. The results showed that, compared with the control, mycelial growth of *N. clavispora* was not significantly inhibited on media containing 1500 mg/L of cyanidin, glycine, luteolin, coniferyl-alcohol, or sinapic acid. In contrast, mycelial growth was significantly inhibited on media containing 1500 mg/L of phloretin or dihydromyricetin (Figure 8; Table S15).

**Figure 9:**
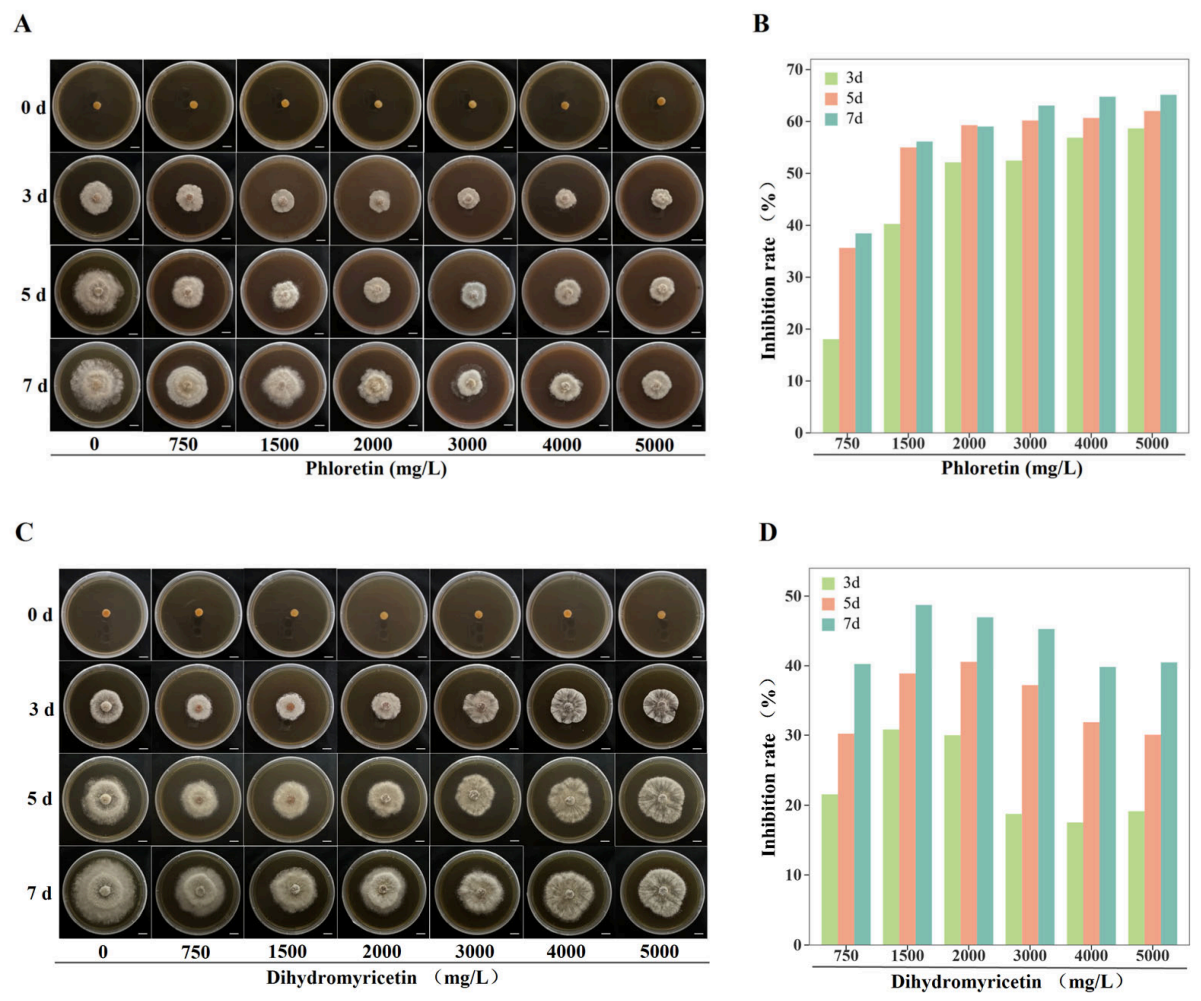
Inhibition of mycelial growth of *N. clavispora* by different concentrations of phloretin and dihydromyricetin. (**A**) Phenotypes of mycelial growth inhibition by phloretin (scale bar = 10 mm); (**B**) inhibition rates of mycelial growth by phloretin; (**C**) phenotypes of mycelial growth inhibition by dihydromyricetin (scale bar = 10 mm); and (**D**) inhibition rates of mycelial growth by dihydromyricetin.

**Figure 10:**
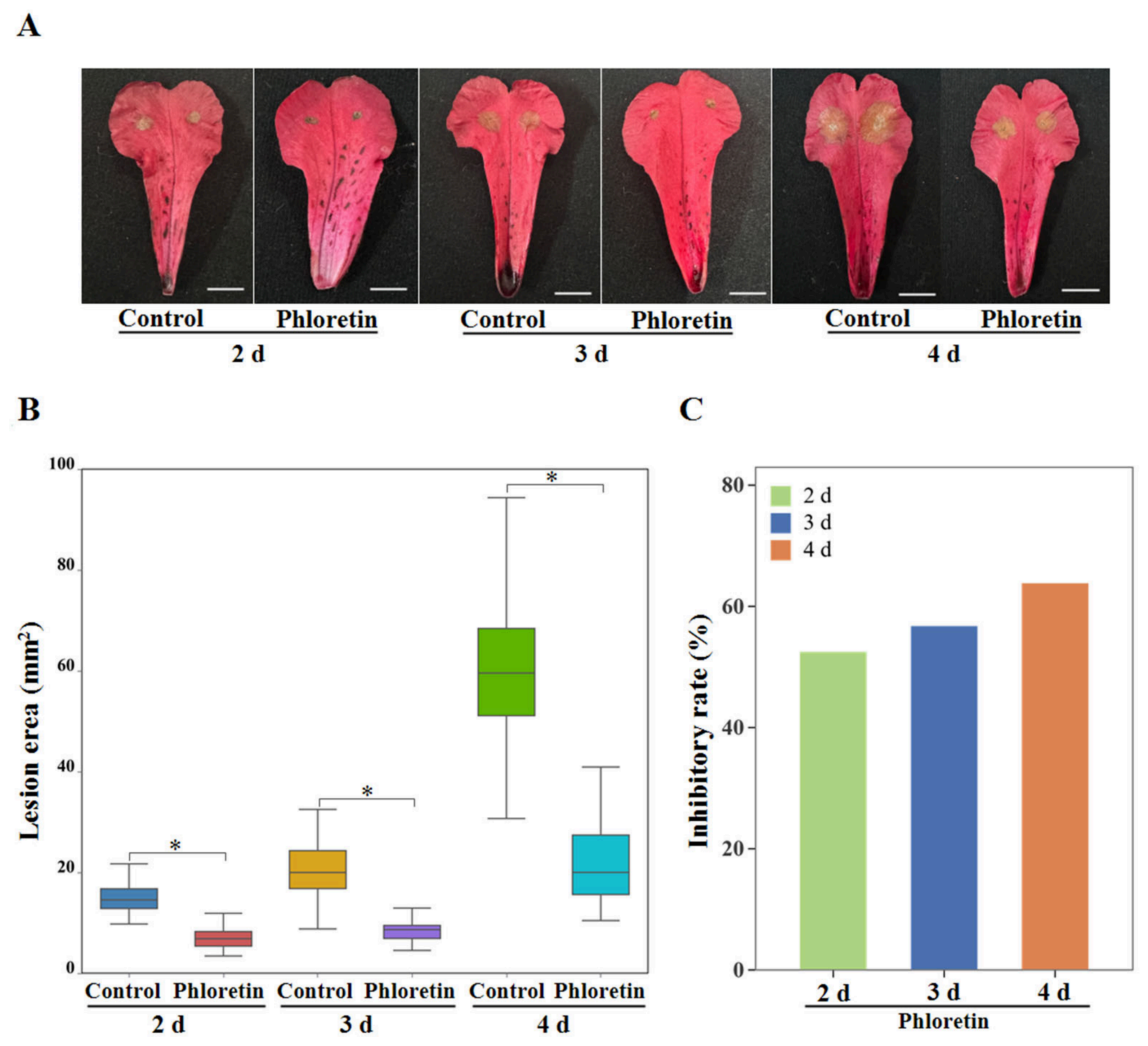
Inhibitory effect of phloretin on petal blight caused by *N. clavispora* in *R. delavayi* petals. (**A**) Lesion phenotypes of petals sprayed with phloretin after inoculation with *N. clavispora.* Control, untreated control; Phloretin, treatment group. Scale bar = 10 mm. (**B**) Lesion areas of petals sprayed with phloretin after inoculation. (**C**) Inhibition rates of lesion area after phloretin application. * indicates significant difference at *p* < 0.05.

Based on the above preliminary screening results, the inhibitory effects of different concentrations of phloretin and dihydromyricetin on mycelial growth of *N. clavispora* were further determined. The results showed that when the concentration of phloretin in the medium exceeded 2000 mg/L, mycelial growth was significantly inhibited (Figure 9A). At a phloretin concentration of 2000 mg/L, the mycelial growth inhibition rates at 3, 5, and 7 days of culture were 52.13%, 59.30%, and 59.03%, respectively (Figure 9B). Therefore, the IC_50_ of phloretin against *N. clavispora* was preliminarily determined to be 2000 mg/L. Exogenous application of 2000 mg/L of phloretin suppressed *N. clavispora* infection on rhododendron petals. At 2, 3, and 4 dpi, the average lesion areas on control petals were 14.77, 20.10, and 60.40 mm^2^, respectively, whereas those of treated petals were 7.02, 8.70, and 21.86 mm^2^ (Figure 10A,B), which were significantly lower than those on the control, with inhibition rates of 52.48%, 56.75%, and 63.81%, respectively (Figure 10C). These results indicate that phloretin can inhibit *N. clavispora* infection on *R. delavayi* petals, and this compound can be recommended as a secondary metabolite for defense against *N. clavispora* causing petal blight in *R. delavayi*. In the concentration gradient set for dihydromyricetin, the highest inhibition rate was observed at 1500 mg/L, with the mycelial growth inhibition rates at 3, 5, and 7 days being 30.81%, 38.85%, and 48.73%, respectively, which did not reach 50% (Figure 9C,D), indicating that dihydromyricetin did not have a significant inhibitory effect on the mycelial growth of *N. clavispora*.

## 3. Discussion

### 3.1. Early Response of R. delavayi Petals to N. clavispora Infection

Phytopathogenic microorganisms typically invade host tissues through stomata, wounds, or mechanical damage and cause cell damage or even necrosis by interfering with host immune responses [49,50,51]. In the present study, no obvious lesions were observed on the mock-inoculated petals, whereas brown lesions appeared as early as 1 dpi after *N. clavispora* infection (Figure 1A), indicating that mechanical wounding alone was insufficient to cause petal blight. The lesion area on infected petals gradually expanded up to 5 dpi (Figure 1B), suggesting that *N. clavispora* exhibits sustained infectivity after entering petal tissues.

In plant–pathogen interactions, rapid production of apoplastic reactive oxygen species (ROS) during the early infection stage is a key event in defending against pathogen invasion [52]. In this study, almost no H_2_O_2_ accumulation was observed in the mock-inoculated or 0 dpi petals, whereas distinct localized brown precipitates appeared at 1 dpi (Figure 2), indicating rapid H_2_O_2_ accumulation at the early stage. Calcium signaling plays an important role in plant–pathogen interactions. After pathogen infection, plants regulate CNGC channels on the cell membrane to activate the expression of downstream CDPK and CaM/CML genes, which participate in ROS accumulation. Moreover, Rboh is a key enzyme catalyzing H_2_O_2_ generation, and rapid apoplastic H_2_O_2_ accumulation mediated by Rboh can directly inhibit or kill invading pathogens [53,54]. In the current study, after *N. clavispora* infection of *R. delavayi* petals, genes encoding CNGCs (*Rhdel01G0286600* and *novel4328*), CALM (*Rhdel02G0004700*, *Rhdel04G0332300*, and *novel2645*), CML (*Rhdel02G0061700*, *Rhdel13G0125300*, *Rhdel11G0128200*, *Rhdel10G0048300*, and *Rhdel09G0038900*), CDPK (*novel1630*, *Rhdel03G0075400*, and *Rhdel12G0202100*), and Rboh (*Rhdel02G0322900*, *Rhdel08G0252500*, and *Rhdel02G0155400*) were significantly upregulated at the early stage (Table S2). This is consistent with the early accumulation of H_2_O_2_, suggesting that the early infection stage may regulate H_2_O_2_ accumulation by inducing the expression of these genes, and this accumulation may directly act against the pathogen. At the early stage of Colletotrichum infection, Arabidopsis thaliana preferentially activates the CNGC- and CDPK-mediated calcium signaling cascade to trigger the basal PTI recognition pathway [55]. Rice also rapidly activates receptor kinases and calcium signals upon early infection by Magnaporthe oryzae, which is highly consistent with the early response pattern observed in *R. delavayi* [56]. Similar results have been reported in *Cucumis sativus* L. [57], *Citrus clementina* [58], and *Vitis pseudoreticulata* [59]. However, excessive H_2_O_2_ accumulation imposes oxidative stress on plants, and plants need to activate ROS-scavenging mechanisms to maintain intracellular ROS homeostasis [60]. *R. delavayi* petals may reduce H_2_O_2_ levels by inducing the expression of peroxidase genes, as evidenced by the significant upregulation of genes encoding peroxidases (*Rhdel13G0276600*, *Rhdel03G0276400*, *Rhdel05G0212800*, and *novel3703*) at the early stage and their identification as important hub genes (Figure 6C). These results indicate that during the early stage of *N. clavispora* infection, *R. delavayi* petals primarily respond to pathogen infection through rapid H_2_O_2_ accumulation and ROS homeostasis.

### 3.2. Middle Response of N. clavispora Petals to R. delavayi Infection

Secondary metabolites are important chemical defense substances utilized by plants to resist pathogen infection [61,62]. Upon recognition of pathogen-associated molecular patterns (PAMPs) by pattern recognition receptors (PRRs), pattern-triggered immunity (PTI) is activated, which, in turn, triggers a series of downstream events, including ROS burst, mitogen-activated protein kinase (MAPK) cascades, and transcription factor activation [63]. In the present study, at the middle stage of *N. clavispora* infection on *R. delavayi* petals, multiple genes involved in PTI signal transduction were significantly upregulated (Table S3), including those encoding pattern recognition receptors EIX1/EIX2 like (*Rhdel03G0230800*, *Rhdel03G0232300*, and *novel2053*), FLS2 (*Rhdel01G0251200*, *Rhdel02G0071200*, *Rhdel07G0134400*, etc.), and the co receptor BAK1 (*Rhdel01G0361000*, *Rhdel13G0185600*, *novel6322*, etc.), as well as the MAPK-cascade-activated MPK3/6 (*Rhdel01G0294100*). Subsequently, activated MPK3/6 phosphorylated multiple transcription factors, including WRKY22 (*Rhdel13G0142100* and *Rhdel13G0226500*), WRKY27 (*Rhdel06G0223200*), WRKY29 (*Rhdel07G0103100*), and WRKY25/33 (*Rhdel01G0319800, Rhdel13G0232300*, etc.). Among these, genes encoding the pattern recognition receptors EIX1/EIX2 like (*Rhdel03G0230800*, *Rhdel03G0232300*, and *novel2053*) and the transcription factor WRKY27 were identified as hub genes at this stage (Figure 6D). The expression of these genes suggests that at the middle stage of *N. clavispora* infection, downstream defense responses are activated, including the expression of pathogenesis-related genes PR1 (*Rhdel11G0226100*), FRK1 (*Rhdel08G0283000*, *Rhdel08G0284500*, and *novel5745*), and the synthesis of secondary metabolites. Upregulation of PR1 and FRK1 has also been reported following infection by various pathogens [24,64].

Secondary metabolites are important compounds in defense against pathogen infection. K-means clustering analysis of differentially accumulated metabolites and KEGG enrichment analysis revealed significant activation of the flavonoid metabolic pathway (Figure 4C). In the flavonoid pathway, genes encoding phenylalanine ammonia lyase (*Rhdel13G0107300*, *Rhdel03G0150000*, and *Rhdel04G0255400*), trans-cinnamate 4-monooxygenase (*Rhdel01G0322300* and *Rhdel13G0066500*), 4-coumarate-CoA ligase (*Rhdel01G0282300*, *Rhdel01G0004700*, and *Rhdel08G0252900*), and CYP75A (*Rhdel03G0045700* and *Rhdel04G0350000*) were significantly upregulated at the middle stage (Figure 7B). Meanwhile, the hub gene encoding 2-oxoglutarate/Fe(II)-dependent oxygenase (*Rhdel03G0305500*) was also significantly upregulated at the middle stage (Figure 6D). These results are consistent with the accumulation of the secondary metabolites phloretin and dihydromyricetin (Figure 7B), indicating that pathogen infection may regulate the accumulation of these secondary metabolites by inducing the expression of these genes. Further antifungal analysis using exogenous phloretin and dihydromyricetin against *N. clavispora* showed that exogenous phloretin significantly inhibited the growth of *N. clavispora* (Figure 9A,B) and also reduced the lesion area on infected petals (Figure 10). This is consistent with the direct antifungal activity of phloretin against various pathogens reported in apple (*Malus domestica* Borkh.) [65,66]. Previous studies have reported that phloretin impairs pathogen cell membranes and disrupts carbohydrate metabolism and energy homeostasis [67,68,69]. Accordingly, we speculate that its antifungal effect against *N. clavispora* also relies on such pathways. Although we demonstrated that phloretin can effectively control flower blight caused by *N. clavispora* under experimental conditions, further field and horticultural investigations are required to optimize application dosage, timing and other parameters. In addition, application costs should also be taken into consideration for practical implementation. In the in vitro antifungal assay using *R. delavayi* petals, dihydromyricetin did not exhibit obvious direct antifungal activity in vitro (Figure 10). It is speculated that dihydromyricetin may indirectly enhance plant disease resistance in vivo by regulating host epigenetic modifications or activating defense-related signaling pathways. This phenomenon has also been reported in the interaction between citrus fruits and *Penicillium italicum* [70].

In this study, the half-maximal inhibitory concentration (IC_50_) of phloretin against *N. clavispora* in vitro was determined to be 2000 mg/L, a value considerably higher than the average endogenous phloretin accumulation level measured by metabolomics in infected petals. However, metabolomic measurements typically reflect the average concentration across entire petal tissues, whereas pathogen infection is often spatially restricted to discrete lesions. It is, therefore, plausible that phloretin undergoes highly localized accumulation in the apoplast or cell walls surrounding infection sites, resulting in substantially higher in situ concentrations than the tissue-averaged levels and, thus, reaching an effective antifungal concentration within the local microenvironment. A similar phenomenon has been observed in sorghum leaves, where the phytoalexins apigeninidin and luteolinidin accumulated as granules specifically at the infection sites of Colletotrichum graminicola, providing direct evidence that local concentrations at the point of pathogen attack can far exceed the bulk tissue averages [71].

### 3.3. Late Response of R. delavayi Petals to N. clavispora Infection

Cell wall remodeling is an important physical barrier in plant defense against pathogen infection. Pathogen infection induces the expression of a series of genes involved in cell wall synthesis and remodeling [72]. In the current study, several hub genes were induced at the late stage of pathogen infection, including those encoding oxidoreductases (*Rhdel10G0051000*, *Rhdel08G0298300*, and *Rhdel11G0156700*), the CRF4 transcription factor (*Rhdel09G0281500*), protein kinases or multidomain transmembrane proteins (*Rhdel01G0360900* and *Rhdel11G0172800*), an E3 ubiquitin ligase gene (*Rhdel03G0079900*), a cellulose synthase-like gene (*Rhdel07G0095800*), and a receptor-like kinase gene (*novel2362*) (Figure 6E). Oxidoreductase functions are involved in catalyzing lignin cross-linking to reinforce the cell wall [73]; protein kinases catalyze cell wall synthesis [74]; E3 ubiquitin ligases regulate callose deposition [75]; cellulose synthases catalyze the synthesis of polysaccharides such as hemicellulose [76,77]; and the CRF4 transcription factor regulates the expression of cell-wall-related genes [78]. The coordinated upregulation of these genes at the late infection stage suggests that *R. delavayi* may participate in the response to pathogen infection through systemic activation of cell-wall-remodeling programs.

Transcriptomic and metabolomic analyses showed that the phenylpropanoid pathway was significantly activated at the late infection stage (Figure S3C; Figure 4D). Phenylpropanoid metabolites often serve as lignin precursors and participate in plant defense responses [79,80]. At the late stage of *N. clavispora* infection on *R. delavayi* petals, sinapic acid, 1-O-sinapoyl-beta-D-glucose, 4-hydroxyphenyl acrylaldehyde, coniferyl-alcohol, and coniferyl-aldehyde significantly accumulated in the phenylpropanoid metabolic pathway. These metabolites act as lignin precursors and participate in the synthesis of lignin monomers [81,82,83,84]. COMT catalyzes the synthesis of sinapic acid and coniferyl-aldehyde, and its encoding genes (*Rhdel01G0282300* and *Rhdel04G0148100*) were also upregulated at the late stage (Figure 7B). In addition, genes encoding enzymes that catalyze the synthesis of 4-hydroxyphenyl acrylaldehyde (*Rhdel07G0331400* and *Rhdel08G0230700*) were also upregulated (Figure 7B), suggesting that they may be involved in the synthesis of 4-hydroxyphenyl acrylaldehyde. Although multiple genes encoding CAD were upregulated (Figure 7B), the accumulation of coniferyl-alcohol was not pronounced at the late stage, possibly because it serves as a substrate for further synthesis of downstream compounds. Furthermore, dihydrokaempferol, kaempferol, luteolin, leucocyanidin, and cyanidin significantly accumulated at the late stage (Figure 7B). Antifungal assays showed that they did not exhibit obvious direct antifungal activity (Figure 10). It is speculated that the accumulation of these metabolites may enhance antioxidant capacity and maintain cell structure stability [85,86].

The synthesis, cycling, and degradation of glutathione (GSH) play central roles in maintaining redox homeostasis and disease resistance by participating in ROS scavenging and signal transduction [87,88]. In the glutathione metabolism pathway, cadaverine, 5-oxoproline, and glycine significantly accumulated at the late infection stage, which was consistent with the upregulated expression of genes encoding GGT1 5 (*Rhdel05G0036900*) and GSS (*Rhdel07G0232000*) (Figure 7B). GSS, a key enzyme in glutathione synthesis, catalyzes the ATP-dependent formation of GSH from γ-glutamylcysteine and glycine in the γ-glutamyl cycle [89]. GGT1-5 is involved in glutathione degradation and recycling, releasing cysteinylglycine by cleaving the γ-glutamyl bond, thereby promoting the regeneration of precursor amino acids for GSH resynthesis [90]. The coordinated expression of GSS and GGT family members may help maintain the dynamic balance of the glutathione pool, thereby enhancing cellular antioxidant capacity during pathogen infection.

In the present study, an excised petal inoculation model was employed, which allowed us to obtain physiological and molecular insights into the petal response to the pathogen. However, several limitations should be considered when interpreting the results. First, the physiological status of excised petals differs from that of petals on intact plants, and such differences may influence both the magnitude and temporal progression of the immune response. Second, the absence of systemic signaling from other organs, such as leaves, in the ex vivo system implies that the whole-plant systemic response to infection cannot be fully recapitulated. Consequently, the stage-specific response patterns observed here are likely representative of local tissue reactions under ex vivo conditions and may not fully reflect the dynamics occurring in intact plants under natural field conditions. Nonetheless, this ex vivo approach remains valuable for the rapid identification of key response determinants and candidate antimicrobial compounds under controlled environments, thereby providing a crucial foundation for subsequent validation in in planta systems. In addition, only three time points were analyzed in this study, and more detailed temporal dynamics remain to be investigated in future work.

## 4. Materials and Methods

### 4.1. Plant Materials and N. clavispora

The *R. delavayi* petals used in this study were derived from plants grown from seeds. The seeds were collected from the Baili Rhododendron Nature Reserve in Guizhou Province, germinated, and cultivated into seedlings in a nursery located in southwestern Guizhou Province (105°28′ E, 26°72′ N; altitude: 1764 m). The seedlings were continuously cultivated in the nursery until flower bud development, after which they were transplanted to a natural-light greenhouse of the Key Laboratory of Plant Physiology and Developmental Regulation, East Campus of Guizhou Normal University, Guiyang, Guizhou Province, China, for further cultivation. After flowering (Figure S4), healthy flowers free of pests and diseases were selected for subsequent *N. clavispora* infection treatment.

The *N. clavispora* strain MR-001 was isolated and purified from infected petals of *Rhododendron agastum* [7]. Previous studies have confirmed that this strain can infect *R. delavayi* petals and cause typical petal blight symptoms [23]. The strain MR-001 is currently preserved in the Key Laboratory of Plant Physiology and Developmental Regulation.

### 4.2. N. clavispora Inoculation and Lesion Analysis

Pathogen inoculation was performed according to a previously established method in our laboratory [7]. The MR-001 strain stored at 4 °C was inoculated onto potato dextrose agar (PDA) medium and cultured at 26 °C for 7 days for activation. The activated colonies were scraped, mixed with sterile water, homogenized using a small sterile homogenizer for 7 min, and adjusted with sterile water to a mycelial suspension concentration of approximately 0.025 g/mL. Disease-free *R. delavayi* flowers were surface-sterilized with 75% ethanol and then inoculated ex vivo. A sterile graver (a blade tip width of approximately 2 mm) was used to gently make a wound of approximately 2 mm in length on the epidermal layer of the middle part of the petal (only cutting the epidermis). An amount of 7 μL of the mycelial suspension was dropped onto the wound in the treatment group, and an equal volume of sterile water was dropped in the control group. The treated flowers were placed in an artificial climate incubator and cultured at 26 °C, 96% relative humidity, and a photoperiod of 16 h light/8 h dark. At 1, 2, 3, 4, and 5 dpi, petals from the control and inoculated groups were excised, flattened, and photographed. The lesion area (including the brownish area and water-soaked margin) was calculated using the ImageJ software (version 1.8.0, National Institutes of Health, Bethesda, MD, USA). The experiment used a completely randomized block design, with 20 petals from different plants per replicate, and four biological replicates (i.e., petals from four different plants).

### 4.3. Histochemical Staining of Reactive Oxygen Species

Histochemical staining was performed according to the method of Daudi and O’Brien (2012), with slight modifications [91]. 3,3′-diaminobenzidine (DAB) staining was used to determine H_2_O_2_ accumulation in petals. Petals at different time points after inoculation were collected, and tissue blocks containing the infection site were excised. The tissue blocks were completely immersed in freshly prepared DAB staining solution (1 mg/mL; pH 3.8), subjected to vacuum infiltration at 0.08 MPa for 30 min, and then incubated at room temperature for 120 min. The staining solution was discarded. Then, 95% ethanol was added, and the petals were decolorized in a water bath at 80 °C, with the 95% ethanol changed every 20 min until complete decolorization. The decolorized petals were rinsed 3–5 times with distilled water, blotted dry on filter paper, and photographed. In addition, petals were subjected to control staining in phosphate-buffered saline (pH 3.8) without DAB. Decolorized petals were observed and photographed using a stereomicroscope (OLYMPUS SZ 61, Hachioji, Japan). Ten petals per biological replicate were selected for staining.

### 4.4. Metabolite Extraction, Detection, and Identification

Lesion area and H_2_O_2_ content were measured daily from 0 to 5 dpi. The lesion area expanded rapidly between 1 and 5 dpi. At 1 dpi, the H_2_O_2_ content reached its first peak, corresponding to the early oxidative burst. At 2 dpi, H_2_O_2_ levels declined, and lesion expansion entered an accelerated phase, indicating the initiation of mid-phase defense responses. By 4 dpi, the lesion area stabilized, and H_2_O_2_ remained at a relatively low level, suggestive of late-stage repair and physiological adaptation. Based on these temporal dynamics, 1, 2, and 4 dpi were selected to represent the early, middle, and late stages of infection, respectively, thereby covering the critical phases of the host defense response. Petal samples from the treatment and control groups at 1, 2, and 4 dpi were selected for metabolite detection. Ten petals from different plants were pooled per replicate, with three biological replicates. The samples were freeze-dried using a Scientz 100F freeze dryer (Scientz Biotechnology Co., Ltd., Ningbo, China) and then ground to a powder using a mill (MM 400, Retsch GmbH, Haan, Germany) at 30 Hz for 1.5 min. An amount of 50 mg of the sample powder was weighed and mixed with 1200 μL of pre-cooled (−20 °C) 70% methanol in water (internal standard extraction solution). The mixture was vortexed for 30 s every 30 min for a total of six times. After centrifugation at 12,000 rpm for 3 min, the supernatant was collected, filtered through a microporous membrane (0.22 μm), and stored in vials for analysis.

The sample extracts were analyzed using a UPLC ESI MS/MS system (UPLC: ExionLC™ AD, SCIEX, Framingham, MA, USA; tandem MS: AB Sciex Triple Quad™ 6500+, SCIEX, Framingham, MA, USA). Separation was performed in an Agilent SB C18 column (2.1 mm × 100 mm; 1.8 μm, Agilent Technologies, Santa Clara, CA, USA). The mobile phase A was water containing 0.1% formic acid, and mobile phase B was acetonitrile containing 0.1% formic acid. The gradient elution program was as follows: 0–9 min, linear change from 95% A to 5% A, and from 5% B to 95% B; 9–10 min, hold at 5% A and 95% B; 10–11.1 min, rapid change from 5% A to 95% A and from 95% B to 5% B; and 11.1–14 min, hold at 95% A and 5% B for column equilibration. The flow rate was 0.35 mL/min; the column temperature was set at 40 °C; and the injection volume was 2 μL. The ESI source’s operating parameters were as follows: a temperature of 500 °C; an ion spray voltage (IS) of 5.5 kV (positive)/−4.5 kV (negative); the ion source gas I (GSI), gas II (GSII), and curtain gas (CUR) set to 50, 60, and 25 psi, respectively; and the collision-induced ionization parameter set to high. Triple-quadrupole (QQQ) scanning was performed using the multiple-reaction monitoring (MRM) mode with the collision gas (nitrogen) set to medium [92].

Missing values were filled with one-fifth of the minimum value of the metabolite, and then the coefficient of variation (CV) of quality control (QC) samples was calculated. Metabolites with CV < 0.5 were retained for data preprocessing. After processing the mass spectrometry data with the Analyst 1.6.3 software, total ion current (TIC) and MRM metabolite detection multi-peak (XIC) chromatograms were obtained. Based on a self-built metabolite database, the retention time and peak area of the detected metabolites were used for qualitative and quantitative analyses. To compare the differences in the content of each metabolite across different samples, the chromatographic peaks of each metabolite detected in different samples were corrected based on retention time and peak shape information to ensure accurate qualitative and quantitative results [93].

### 4.5. Screening of Differentially Accumulated Metabolites (DAMs) and K-Means Clustering Analysis

Principal component analysis (PCA) was performed using the base package in the R software (v4.3.1), hierarchical cluster analysis (HCA) was performed using the ComplexHeatmap package, and Pearson correlation coefficient analysis was performed using the base package to evaluate sample differences and reproducibility [94]. After Log2 transformation and mean centering, orthogonal partial least-squares discriminant analysis (OPLS DA) was performed using the R software. DAMs were screened based on variable importance in projection (VIP) derived from the OPLS DA model and fold change (FC), with the following criteria: VIP > 1 and |Log_2_FC| ≥ 1.0. K-means clustering analysis of DAMs was performed using the R software. Functional annotation and enrichment analysis of DAMs were performed using the Kyoto Encyclopedia of Genes and Genomes (KEGG) compound database (http://www.kegg.jp/kegg/compound/) (accessed on 15 September 2025) and the KEGG Pathway database (http://www.kegg.jp/kegg/pathway.html) (accessed on 15 September 2025) [95].

### 4.6. RNA Extraction, Library Construction, RNA Sequencing, and Differential Expression Gene Analysis

Consistent with the metabolome samples, petal samples from the treatment and control groups at 1, 2, and 4 dpi were selected for transcriptome sequencing. Ten petals from different plants were pooled per replicate, with three biological replicates. Total RNA was extracted from petals using a CTAB reagent combined with ethanol precipitation. RNA quality was assessed using a NanoDrop One spectrophotometer (NanoDrop Technologies, Wilmington, DE, USA), a Qubit 3.0 Fluorometer (Life Technologies, Carlsbad, CA, USA), and electrophoresis to ensure that the RNA quality met sequencing requirements.

cDNA sequencing libraries were constructed according to the VAHTS Universal V6 RNA seq Library Prep Kit for MGI^®^ (Cat. No. NRM605, Vazyme Biotech Co., Ltd., Nanjing, China) manual. After quality validation, the libraries were subjected to paired-end sequencing on a DNBSEQ T7 sequencer. Sequencing was completed at Wuhan Benagen Technology Co., Ltd. (Wuhan, China). After sequencing, raw reads were processed with fastp to remove low-quality reads and adapter sequences, yielding clean reads [96]. Quality control of the filtered data was performed using fastqc [97]. Using the *R. delavayi* genome (http://bioinfor.kib.ac.cn/RPGD/download_genome.html, accessed on 15 September 2025) as the reference, clean reads were aligned to the reference genome using STAR (version 2.7.9a) with the following parameters: —outFilterMultimapNmax 10, —outFilterMismatchNmax 10, —outFilterMismatchNoverLmax 0.3, —alignIntronMin 20, —alignIntronMax 1,000,000, —alignMatesGapMax 1,000,000, —outFilterScoreMinOverLread 0.66, and —outFilterMatchNminOverLread 0.66. After alignment, only uniquely mapped reads were retained for subsequent gene expression quantification, and multi-mapped reads were excluded from the count matrix [91]. Transcript assembly and novel gene prediction were performed using StringTie (accessed on 15 September 2025) [98,99]. Based on the alignment results and gene position information on the reference genome, read counts for each gene were calculated using the RSEM software (accessed on 15 September 2025), and gene expression levels were normalized as fragments per kilobase of transcript per million mapped reads (FPKM) [100]. Differentially expressed genes (DEGs) were screened with the following criteria: |log_2_ fold change| > 1 and false discovery rate (FDR) < 0.05 [101].

### 4.7. WGCNA and KEGG Enrichment Analysis of DEGs

All DEGs identified at 1, 2, and 4 dpi were selected for weighted gene co-expression network analysis (WGCNA) using the “WGCNA” package in the R software (v4.3.1) to identify modules significantly associated with pathogen infection [102]. Genes in the significant modules were imported into the Cytoscape software (v1.7.251; https://cytoscape.org/index.html) (accessed on 26 January 2026) for visualization and construction of gene co-expression networks. Gene Ontology (GO) and KEGG pathway enrichment analyses of DEGs were performed using the Metware platform (https://cloud.metware.cn/). Significantly enriched GO terms and KEGG pathways were identified with a threshold of *p* < 0.05 [95]. Pearson correlation analysis was performed to calculate correlation coefficients between the FPKM expression values of hub genes and the relative contents of differentially accumulated metabolites. Gene–metabolite pairs with |r| ≥ 0.70 and *p* < 0.05 were screened out, and hierarchical clustering was conducted for both rows and columns before generating the correlation heatmap.

### 4.8. Inhibition of N. clavispora Mycelial Growth by Metabolites

Candidate metabolites with potential antifungal activity were selected (all purchased from Beijing Solarbio Science & Technology Co., Ltd., Beijing, China; https://www.solarbio.com/) (accessed on 29 January 2026). Referring to the method of Shi X et al. (2024) [23] with slight modifications, the above metabolites were prepared as 1500 mg/L solutions, filtered through 0.22 μm sterile filters, and evenly spread on the surface of PDA medium. Mycelial plugs (8 mm in diameter) taken from activated cultures were inoculated onto the prepared media. When dissolving metabolites using 1% DMSO solution, we found that certain metabolites at high concentrations required a small amount of 1% sodium hydroxide for complete dissolution. For this reason, 1% DMSO and 1% NaOH were selected as cosolvents. Accordingly, a solution containing 1% NaOH and 1% DMSO was used as the solvent control, while pure PDA medium served as the blank control. The cultures were incubated at 28 °C, and colonies were photographed at 0, 3, 5, and 7 days. The colony area was measured using the ImageJ software (version 1.8.0), with five biological replicates per treatment. Statistical analysis was performed using IBM SPSS Statistics (version 27) to screen for metabolites with potential antifungal effects. Based on the screening results, the half-maximal inhibitory concentration (IC_50_) was further determined. Subsequently, PDA media containing phloretin or dihydromyricetin at concentrations of 750, 1500, 2000, 3000, 4000, and 5000 mg/L were prepared using the same method, with five biological replicates per treatment. Each treatment had five biological replicates. Mycelial plugs were inoculated and photographed, and colony areas were measured as described above. The mycelial inhibition rate was calculated using the following formula: Inhibition (%) = [(Dc − Dt)/Dc] × 100, where Dc = average colony area in the control group, and Dt = average colony area in the treatment group [103].

### 4.9. Suppression of N. clavispora Infection in R. delavayi Petals by Metabolites

Following the petal inoculation method described above, petals were inoculated with *N. clavispora* mycelial suspension. After 24 h, petals were sprayed every 12 h with either a 1% DMSO solution or a 2000 mg/L phloretin solution. The petals were photographed at 2, 3, and 4 days after inoculation, and lesion area was measured using the ImageJ software (version 1.8.0). Each treatment had four biological replicates, with 20 petals per replicate. The inhibition rate was calculated according to the following formula: Inhibition (%) = [(Dc − Dt)/Dc] × 100, where Dc = average lesion area of control petals, and Dt = average lesion area of treated petals [26].

### 4.10. Statistical Analysis

Data are presented as means ± standard deviation (SD). One-way analysis of variance (ANOVA) followed by the least-significant difference (LSD) multiple-comparison test was used, and *p* < 0.05 was considered statistically significant.

## 5. Conclusions

**Figure 11:**
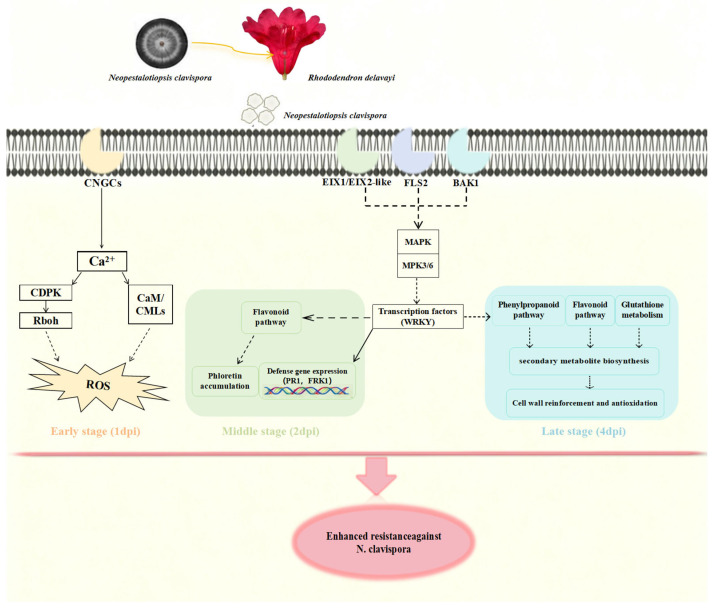
Schematic diagram of the disease resistance mechanism in *R. delavayi* petals in response to *N. clavispora* infection. CNGCs: cyclic nucleotide-gated channels; CDPK: calcium-dependent protein kinase; Rboh: respiratory burst oxidase homolog; CaM: calmodulin; CMLs: calmodulin-like proteins; ROS: reactive oxygen species; EIX1/EIX2-like: ethylene-inducing xylanase 1/2-like; FLS2: flagellin-sensing 2; BAK1: BRI1-associated kinase 1; MAPK: mitogen-activated protein kinase; MPK3/6: mitogen-activated protein kinase 3/6; PR1: pathogenesis-related protein 1; FRK1: Flg22-induced receptor-like kinase 1.

Combined widely targeted metabolomic and transcriptomic profiling revealed a tiered, stage-specific immune regulatory network in *R. delavayi* petals during *N. clavispora* infection, a core novelty of this study. Calcium–ROS signaling mediates early defense; PTI signaling and phloretin accumulation dominate mid-stage resistance, while phenylpropanoid, flavonoid and glutathione metabolism reprogramming strengthens late-stage cell wall and antioxidant defense (Figure 11). In vitro bioassays validated phloretin’s strong inhibitory effect on rhododendron petal blight, providing a natural bioactive compound resource for sustainable rhododendron disease management. To further interpret and extend the findings obtained in this study, our subsequent research will focus on three directions: field evaluation of the practical disease-suppressive efficacy of phloretin, systematic dissection of its intrinsic antifungal molecular mechanism, and genetic functional validation of the core hub defense genes identified in the present work.

## Figures and Tables

**Table 1:** Differentially accumulated metabolites and their reported antifungal activities based on the published literature.

Metabolites	Chemical Formula	Antifungal Activity	References
Kaempferol	C_15_H_10_O_6_	*Candida parapsilosis*	[38]
Cyanidin	C_31_H_29_O_14_	*Proteobacteria*	[39]
Leucocyanidin	C_15_H_14_O	/	/
Dihydrokaempferol	C_15_H_12_O_6_	/	/
Dihydromyricetin	C_15_H_12_O_8_	*Aspergillus fumigatus*	[40]
Luteolin	C_15_H_10_O_6_	*Botrytis cinerea/Botrytis cinerea/Penicillium expansum*	[41,42]
Phloretin	C_15_H_14_O_5_	*Colletotrichum gloeosporioides*	[43]
Sinapic acid	C_11_H_12_O_5_	*Fusarium*	[44]
1-O-sinapoyl-beta-D-glucose	C_17_H_22_O_10_	/	/
4-hydroxyphenyl acrylaldehyde (p-coumaraldehyde)	C_9_H_8_O_3_	*Rhizoctonia solani/Magnaporthe grisea/Ustilaginoidea virens*	[45]
Coniferyl-aldehyde	C_9_H_8_O_3_	*Dactylobotrys graminicola*	[46]
Coniferyl-alcohol	C_10_H_12_O_3_	*Neofusicoccum parvum/Dothiorella viticola/Diplodia seriata*	[47]
Cadaverine	C_5_H_14_N_2_	/	/
5-oxoproline	C_5_H_7_NO_3_	/	/
Glycine	C_18_H_35_NO_3_	*Penicillium citrinum*	[48]
L-glutamate	C_5_H_9_NO_4_	/	/

## Data Availability

The transcriptome sequencing data have been deposited in the NCBI BioProject database under accession number PRJNA1321767. Other datasets generated and/or analyzed during the current study are available from the corresponding author upon reasonable request.

## Supplementary Materials

The following supporting information can be downloaded at https://www.mdpi.com/article/10.3390/plants15152362/s1. Figure S1: Overlay of total ion current (TIC) chromatograms of quality control (QC) samples in mass spectrometry detection. The high overlap of the TIC curves, as reflected by consistent retention times and peak intensities, indicates excellent signal stability of the mass spectrometer across different injection times for the same sample, thereby ensuring data reproducibility and reliability. (A) Negative ion mode; (B) positive ion mode. Figure S2: Pathway enrichment analysis (top 10) of all differentially accumulated metabolites (DAMs) detected at 1, 2, and 4 dpi. (A) IRd1d_vs_MRd1d_KEGG_Enrichment; (B) IRd2d_vs_MRd2d_KEGG_Enrichment; and (C) IRd4d_vs_MRd4d_KEGG_Enrichment. Figure S3: GO enrichment analysis (top 10) and KEGG enrichment analysis of differentially expressed genes (DEGs) at 1, 2, and 4 dpi. (A) GO enrichment analysis of IRd1d_vs_MRd1d; (B) GO enrichment analysis of IRd2d_vs_MRd2d; (C) GO enrichment analysis of IRd4d_vs_MRd4d; (D) KEGG enrichment analysis of IRd1d_vs_MRd1d; (E) KEGG enrichment analysis of IRd2d_vs_MRd2d; and (F) KEGG enrichment analysis of IRd4d_vs_MRd4d; Figure S4: Morphological characteristics of *R. delavayi.* (A) Whole-potted seedling plant. Scale bars = 10 mm; (B) inflorescence (scale bars = 300 mm); and (C) single flower (scale bars = 10 mm). Figure S5. Pearson correlation analysis between the expression of key genes and the relative accumulation of differential metabolites. Pearson correlation coefficients were calculated, and gene-metabolite pairs satisfying |r| ≥ 0.70 and *p* < 0.05 were retained. Hierarchical clustering was performed on both rows and columns. The color gradient represents the correlation coefficient value; red indicates positive correlation, green indicates negative correlation. Table S1: A total of 326 DAMs identified in *R. delavayi* petals at 1 dpi with *N. clavispora*. Table S2: A total of 240 DAMs identified in *R. delavayi* petals at 2 dpi with *N. clavispora*. Table S3: A total of 362 DAMs identified in *R. delavayi* petals at 4 dpi with *N. clavispora*. Table S4: Significantly enriched KEGG pathways of DAMs in *R. delavayi* petals at 1 dpi with *N. clavispora*. Table S5: Significantly enriched KEGG pathways of DAMs in *R. delavayi* petals at 2 dpi with *N. clavispora*. Table S6: Significantly enriched KEGG pathways of DAMs in *R. delavayi* petals at 4 dpi with *N. clavispora*. Table S7: K-means clustering analysis of DAMs from *R. delavayi* petals infected with *N. clavispora*. Table S9: Summary of RNA-Seq data quality and read mapping statistics for *R. delavayi* petal samples. Table S9: DEGs identified in *R. delavayi* petals at 1 dpi with *N. clavispora*. Table S10: DEGs identified in *R. delavayi* petals at 2 dpi with *N. clavispora*. Table S11: DEGs identified in *R. delavayi* petals at 4 dpi with *N. clavispora*. Table S12: KEGG enrichment pathways of DEGs in *R. delavayi* petals at 1 dpi with *N. clavispora*. Table S13: KEGG enrichment pathways of DEGs in *R. delavayi* petals at 2 dpi with *N. clavispora*. Table S14: KEGG enrichment pathways of DEGs in *R. delavayi* petals at 4 dpi with *N. clavispora*. Table S15: Effects of different differential secondary metabolites at 1500 mg/L on mycelial growth area on *N. clavispora*.
