# Supplementary material for: Metabolomic and Transcriptomic Responses of Rhododendron delavayi Petals Infected with Neopestalotiopsis clavispora at Different Stages
Source: Plants (Basel). 2026 Jul 31;15(15):2362. doi: 10.3390/plants15152362 (PMC13468463; doi:10.3390/plants15152362)
Supplement: Supplementary file 1 [file plants-15-02362-s001.zip › Supplementary Figure.pdf]

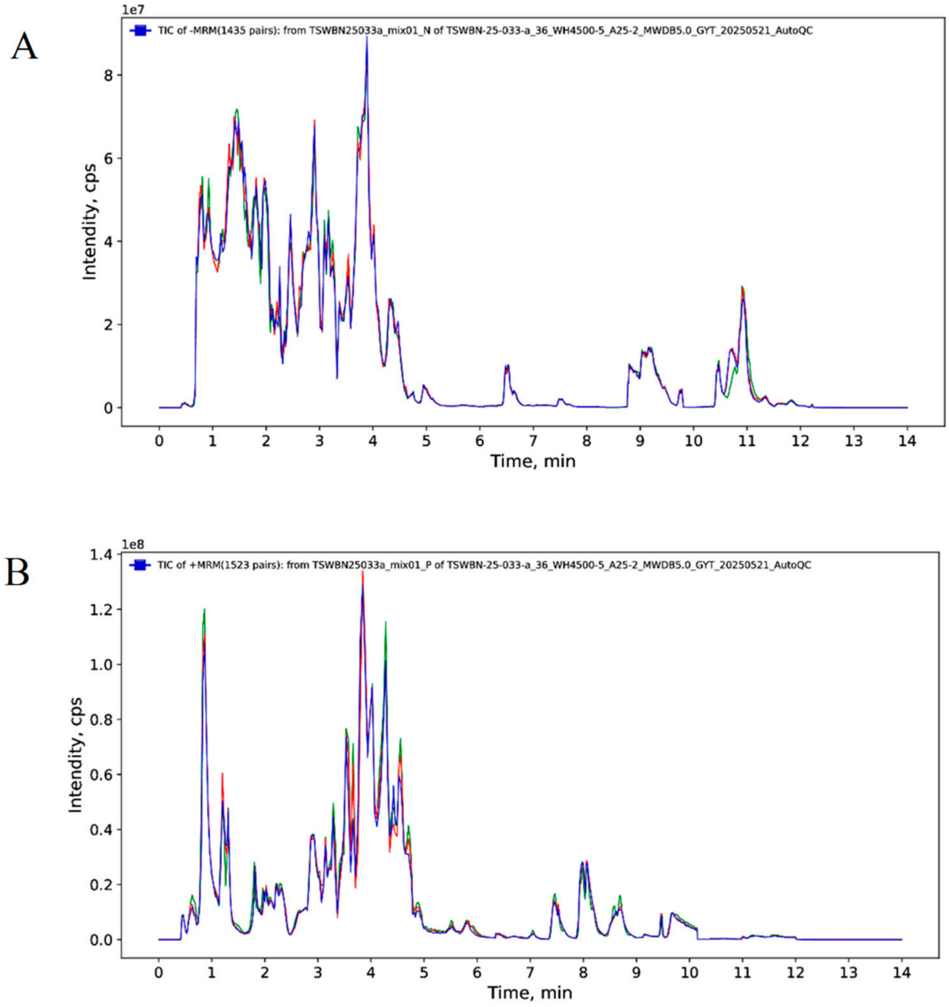

Figure S1. Overlay of total ion current (TIC) chromatograms of quality control (QC) samples in mass spectrometry detection. The high overlap of the TIC curves, as reflected by consistent retention times and peak intensities, indicates excellent signal stability of the mass spectrometer across different injection times for the same sample, thereby ensuring data reproducibility and reliability. (A) Negative ion mode; (B) Positive ion mode.

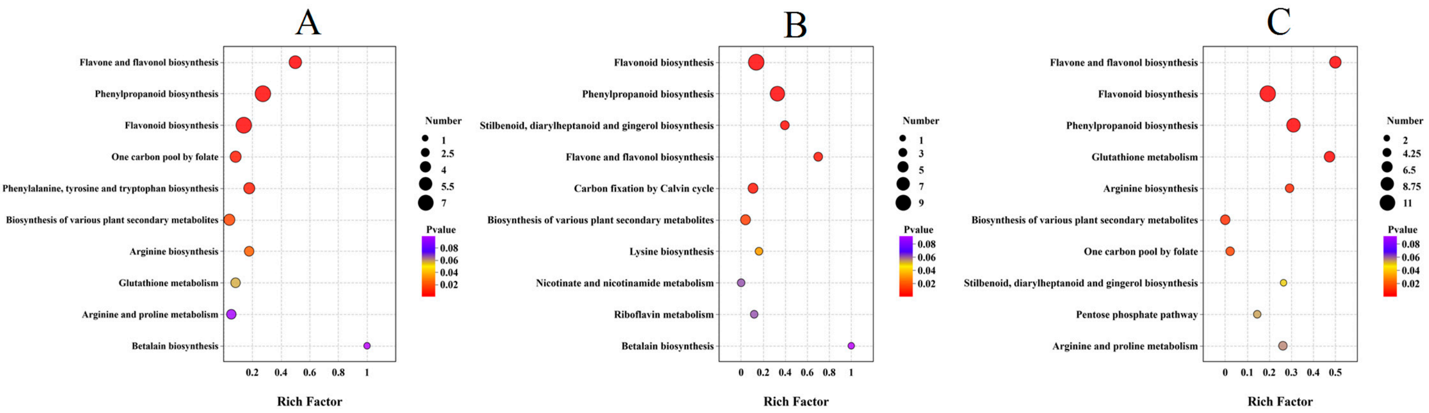

Figure S2. Pathway enrichment analysis (Top 10) of all differentially accumulated metabolites (DAMs) detected at 1, 2, and 4dpi.

(A) IRd1d_vs_MRd1d_KEGG_Enrichment; (B) IRd2d_vs_MRd2d_KEGG_Enrichment; (C) IRd4d_vs_MRd4d_KEGG_Enrichment.

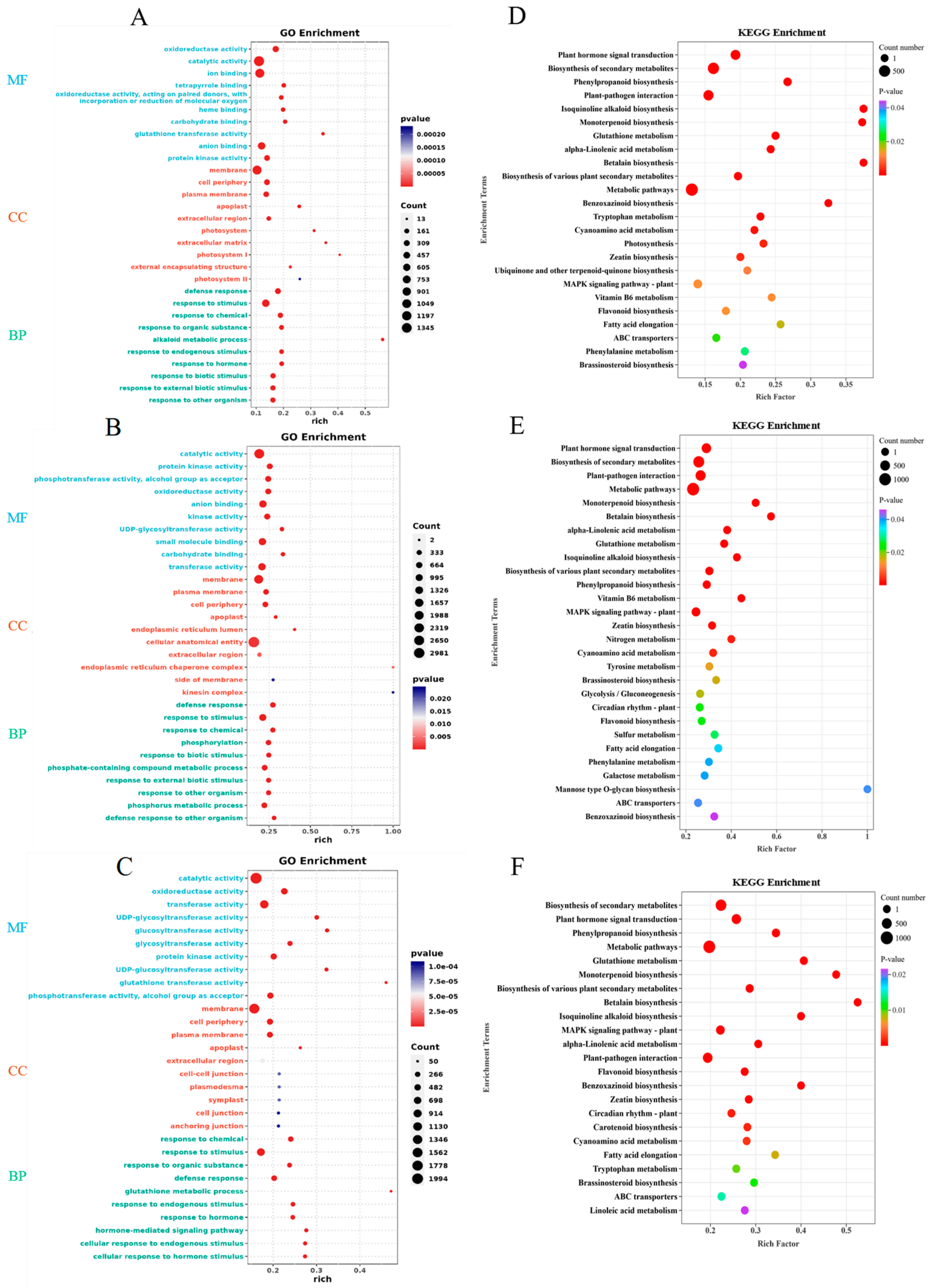

Figure S3. GO enrichment analysis (Top 10) and KEGG enrichment analysis ($P < 0.05$) of differentially expressed genes (DEGs) at 1, 2, and 4 dpi. (A) GO enrichment analysis of IRd1d_vs_MRd1d; (B) GO enrichment analysis of IRd2d_vs_MRd2d; (C) GO enrichment analysis of IRd4d_vs_MRd4d; (D) KEGG enrichment analysis of IRd1d_vs_MRd1d; (E) KEGG enrichment analysis of IRd2d_vs_MRd2d; (F) KEGG enrichment analysis of IRd4d_vs_MRd4d.

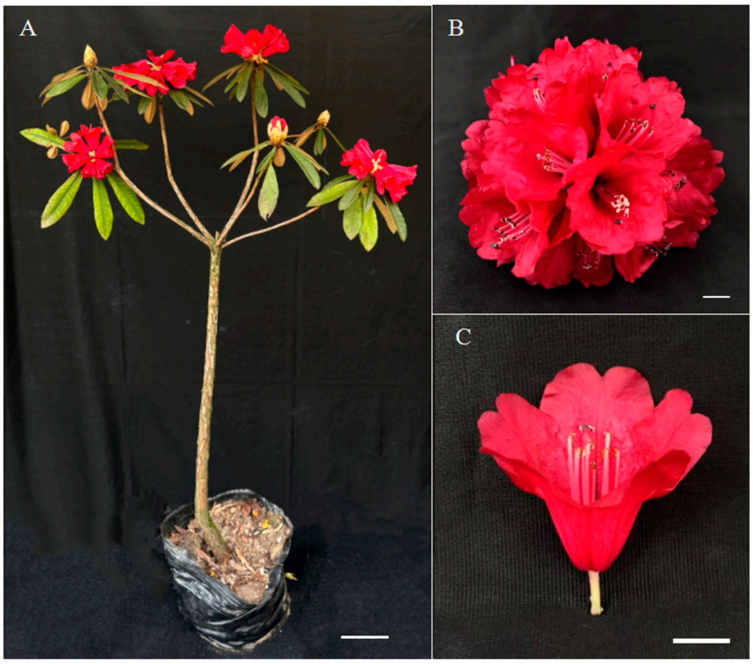

Rhododendron delavayi

Figure S4. Morphological characteristics of Rhododendron delavayi. (A)Whole potted seedling plant. Scale bars=10mm; (B) Inflorescence. Scale bars=300mm; (C) Single flower. Scale bars=10mm.

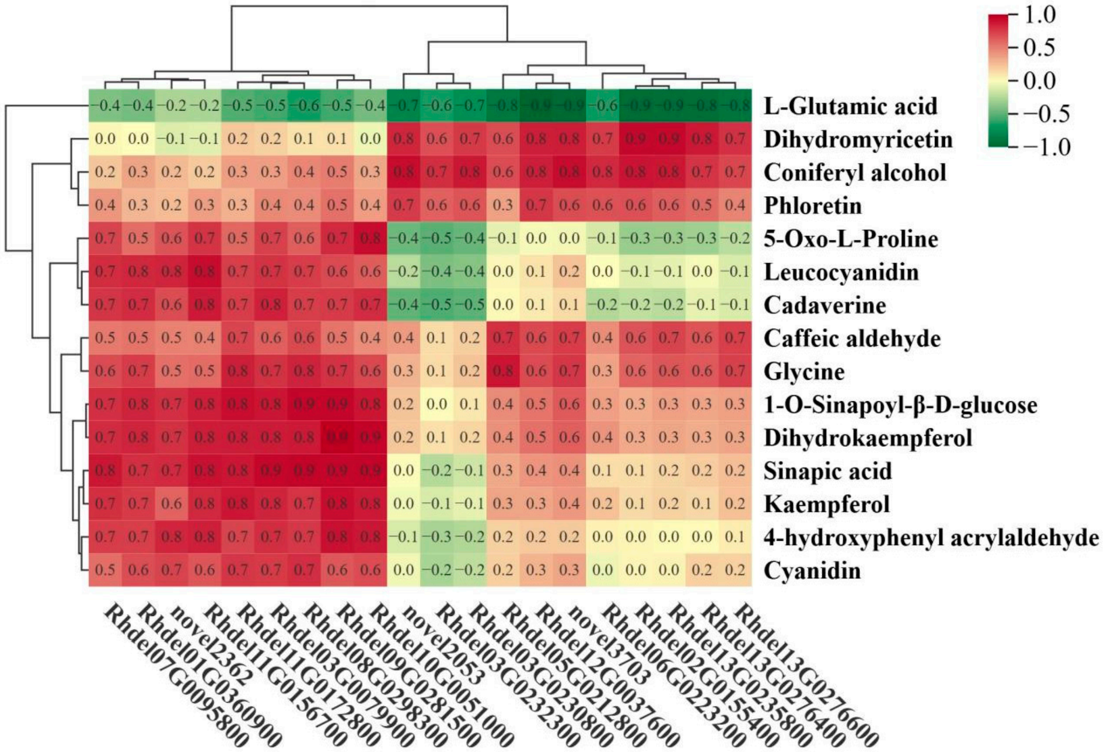

Figure S5. Pearson correlation analysis between the expression of key genes and the relative accumulation of differential metabolites. Pearson correlation coefficients were calculated, and gene-metabolite pairs satisfying $|r| \geq 0.70$ and $P < 0.05$ were retained. Hierarchical clustering was performed on both rows and columns. The color gradient represents the correlation coefficient value; red indicates positive correlation, green indicates negative correlation.
